# The Principal Forces of Oocyte Polarity Are Evolutionary Conserved but May Not Affect the Contribution of the First Two Blastomeres to the Blastocyst Development in Mammals

**DOI:** 10.1371/journal.pone.0148382

**Published:** 2016-03-31

**Authors:** Sayyed-Morteza Hosseini, Fariba Moulavi, Nima Tanhaie-Vash, Vajihe Asgari, Hamid-Reza Ghanaei, Maryam Abedi-Dorche, Naser Jafarzadeh, Hossein Gourabi, Abdol-Hossein Shahverdi, Ahmad Vosough Dizaj, Abolfazl Shirazi, Mohammad-Hossein Nasr-Esfahani

**Affiliations:** 1 Department of Reproductive Biotechnology, Reproductive Biomedicine Research Center, Royan Institute for Biotechnology, ACECR, Isfahan, Iran; 2 Department of Medical Physics, Tarbiat Modares University, Tehran, Iran; 3 Department of Genetics at Reproductive Biomedicine Research Center, Royan Institute for Reproductive Biomedicine, ACECR, P.O. Box: 19395–4644, Tehran, Iran; 4 Department of Embryology at Reproductive Biomedicine Research Center, Royan Institute for Reproductive Medicine, ACECR, Tehran, Iran; 5 Department of Reproductive Imaging at Reproductive Biomedicine Research Center, Royan Institute for Reproductive Biomedicine, ACECR, Tehran, Iran; 6 Reproductive Biotechnology Research Center, Avicenna Research Institute, ACECR, Tehran, Iran; 7 Research Institute of Animal Embryo Technology, Shahrekord University, Shahrekord, Iran; Institute of Zoology, Chinese Academy of Sciences, CHINA

## Abstract

Oocyte polarity and embryonic patterning are well-established features of development in lower species. Whether a similar form of pre-patterning exists in mammals is currently under hot debate in mice. This study investigated this issue for the first time in ovine as a large mammal model. Microsurgical trisection of unfertilized MII-oocytes revealed that cortical cytoplasm around spindle (S) contained significant amounts of total maternal mRNAs and proteins compared to matched cytoplast hemispheres that were located either near (NS) or far (FS) -to-spindle. RT-qPCR provided striking examples of maternal mRNA localized to subcellular substructures S (*NPM2*, *GMNN*, *H19*, *PCAF*, *DNMT3*A, *DNMT1*, and *STELLA*), NS (*SOX2*, *NANOG*, *POU5F1*, and *TET1*), and FS (*GCN*) of MII oocyte. Immunoblotting revealed that specific maternal proteins DNMT3A and NANOG were asymmetrically enriched in MII-spindle-half of the oocytes. Topological analysis of sperm entry point (SEP) revealed that sperm preferentially entered via the MII-spindle-half of the oocytes. Even though, the topological position of first cleavage plane with regard to SEP was quite stochastic. Spatial comparison of lipid content revealed symmetrical distribution of lipids between 2-cell blastomeres. Lineage tracing using Dil, a fluorescent dye, revealed that while the progeny of leading blastomere of 2-cell embryos contributed to more cells in the developed blastocysts compared to lagging counterpart, the contributions of leading and lagging blastomeres to the embryonic-abembryonic parts of the developed blastocysts were almost unbiased. And finally, separated sister blastomeres of 2-cell embryos had an overall similar probability to arrest at any stage before the blastocyst (2-cell, 4-cell, 8-cell, and morula) or to achieve the blastocyst stage. It was concluded that the localization of maternal mRNAs and proteins at the spindle are evolutionarily conserved between mammals unfertilized ovine oocyte could be considered polar with respect to the spatial regionalization of maternal transcripts and proteins. Even though, the principal forces of this definitive oocyte polarity may not persist during embryonic cleavages.

## Introduction

Oocyte polarity and embryonic patterning are well-established features of development in lower species [1]. However, a long-standing question in mammalian development is how the embryonic-abembryonic axis of the blastocyst is first established. The answer of this question is crucially important to know whether the cleavage divisions that regulate embryonal axis formation are pre-patterned [2, 3] or affect the establishment of totipotency [4] in mammalian development. At present, three classic models exist to explain the mechanism of early cell lineage specification in mammals: the “pre-patterning”, the “inside-outside” and the “cell polarity” [3]. In pre-patterning model, analogous to the mechanism that exists in *Drosophila*, *C*. *elegans* and *Xenopus*, determinants of embryonal axis formation are anticipated either during oocyte maturation or upon fertilization [5]. The inside-outside model denotes the importance of topological cell position at morula stage on the blastocyst patterning [6]. And, the cell polarity model denotes the importance of apical and distal membrane domains appear at 8-to-16 cell embryo stage on fate specification of descendant embryonic cells [7].

The underlying mechanisms of embryonal axis formation are incompletely understood in mammals. In mouse, as the most studied system, the mechanism of first lineage commitment is currently under hot debate. A number of studies believe that mouse cell fate is pre-patterned and the descendant embryonic cells arising from the first cleavage division have distinguishable fates [5, 8, 9, 10, 11]. In contrast, other studies believe that the order of cleavage does not determine the allocation of cells in embryonic-abembryonic regions [12, 13, 14, 15, 16, 17, 18, 19]. The asymmetric localization of maternal transcripts is an essential polarity determinant, directing cis-regulation of zygotic genes in metazoan [20, 21, 22]. Although conservation of this developmental mechanism in mammals is assumed, recent reports are conflicting with arguments both for [1, 4, 21, 23, 24, 25] and against [16, 26] the idea of oocyte polarity in mouse. Notably, a number of studies demonstrated that the fertilizing sperm have a preferential entry point (SEP) into the oocyte in mice [10, 16]. Even though, a subsequent study by the latter group provided evidence that the space asymmetry exerted by the first polar body and the zona pellucida directs SEP [18]. If transcriptional asymmetry does exist within the unfertilized mammalian oocytes, it is possible that the mechanism of embryonal axis formation in higher mammals is established before fertilization when the oocyte becomes polarized.

Why is the study of embryo patterning important? For many years, it has been recommended that sperm should be injected far from the first polar body during intacytopalsmic sperm injection (ICSI) [27]. Somatic cell nuclear transfer (SCNT) is carried out by removing part of ooplasm that encompasses MII-chromosomes [28]. Monozygotic twining is of great potential implications for the multiplication of elite animals and endangered animals [29]. Recently, monozygotic twining using small molecules provided promising opportunity for splitting an embryo into two parts: a part that will develop to term and the other part that could be cultured in vitro to generate autologous embryonic stem cell (ESC) for its owner [30, 31]. Preimplantation genetic diagnosis (PGD) is increasingly used to screen possible genetic diseases or chromosome abnormalities of embryos. In routine PGD procedure, one (or more) blastomere is biopsied from a day 3 embryo [32]. All these techniques have been established based on a long-lasting concept that in mammals, the oocyte is symmetric and the balstomeres of early embryo are equal in their competences. Even though, if further studies could support the existence of cytoplasmic “polarity” of oocyte and “inequality” of early blastomeres, major refinements/modifications may be required for these manipulative/diagnostic techniques [33].

Lineage-tracing of cleavage stage embryos is poorly understood in mammalian species rather than mice, with only two studies assessed embryo patterning in porcine [34, 35]. However, choosing the adequate animal model to test biological hypotheses is still an open challenge and increasing evidence suggests that mouse may not be a relevant model for human biological studies. Given the current knowledge of embryonal axis formation in mice and owing the close similarities exist between human and sheep embryos in terms of metabolism and key stages of pre and post-implantation development [36, 37, 38], we have used ovine as an animal model to evaluate the likelihood of oocyte polarity and embryo patterning in mammalian species.

## Materials and Methods

Unless otherwise specified, all chemicals and media were obtained from Sigma Chemical Co. (St. Louis, MO, USA) and Gibco (Grand Island, NY, USA), respectively. All procedures undertaken in this study were reviewed and approved by Institutional Animal Care and Use Committee (IACUC) of Royan Institute.

### Ovine oocyte preparation and embryo production

The procedures used for oocyte preparation and in vitro embryo production were as described previously [28]. In brief, a total of 2308 ovaries of local breed adult ewes (mainly from Afshari and Naieni breeds, aged between 9 months to 7 years old) were obtained from two local abattoirs (Khomeinishahr and Kashan cities) in Isfahan province, Antral follicles (2–6 mm diameter) of ovaries were aspirated to obtain cumulus-oocyte complexes (COCs). Selected COCs with homogenous cytoplasm and more than three layers of surrounding cumulus cells were washed three times in hepes-buffered tissue culture medium 199 (HTCM199) + 10% sheep serum (SS) followed by three further washings in maturation medium (TCM199 containing 2.5 mM Na-pyruvate, 1 mM L-glutamine, 100 IU/ml penicillin, 100 μg/ml streptomycin, 10% SS, 10 μg/ml ovine FSH, 10 μg/ml ovine LH, 1 μg/ml estradiol-17ß, and 0.1 mM cysteamine). Oocytes were then cultured for 20–22 h in groups of 20–25 in 100 μl droplets of maturation medium covered with mineral oil at 38.5°C, 5% CO_2_ and humidified air. Matured COCs were then used for embryo development and presumptive zygotes were cultured in groups of 6–8 in 20 μl droplets of a modified formulation of synthetic oviduct fluid described (mSOF) at 39°C, 6% CO_2_, 5% O_2_ and humidified air for 168 h [39].

### Spatial distribution of maternal mRNA within MII-oocytes

**Fig 1:**
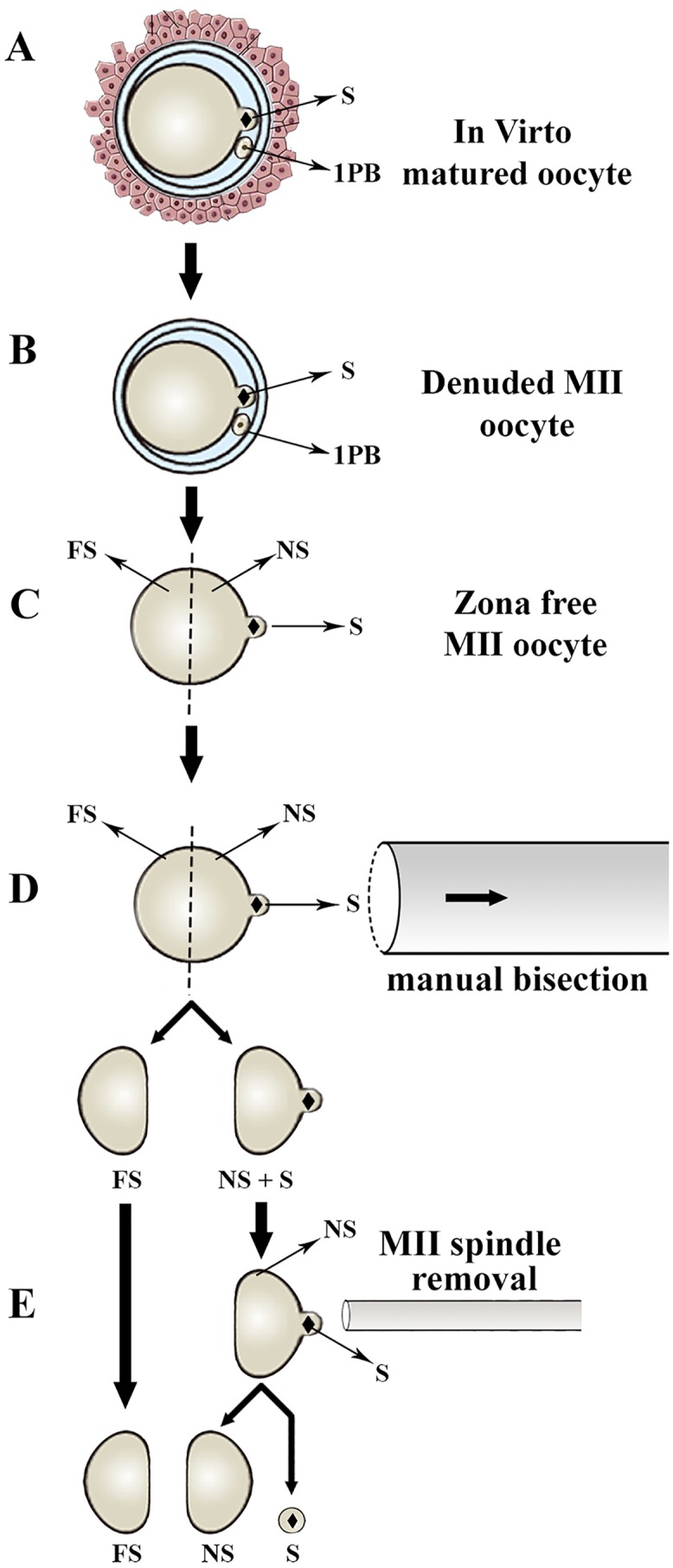
Schematic illustration of the procedure used for microsurgical trisection of ovine unfertilized MII-oocytes using a manual method of oocyte trisection. A, B) In vitro matured oocytes were denuded of cumulus cells and oocytes with evident first polar body (1Pb) were selected for zona removal. C) Ovine oocytes partially extrude the MII-spindle and associated chromosomes (S) as a cytoplasmic protrusion which is particularly evident following zona removal. D) Using this cytoplasmic protrusion as a reference point, oocytes were first bisected to oocyte hemispheres that were either near-to (NS) or far-from (FS) the MII-spindle (S). E) The obtained NS oocyte hemispheres were then used for preparation of cortical materials containing MII-chromosomes (S) and the enucleated NS oocyte hemispheres.

**Fig 2:**
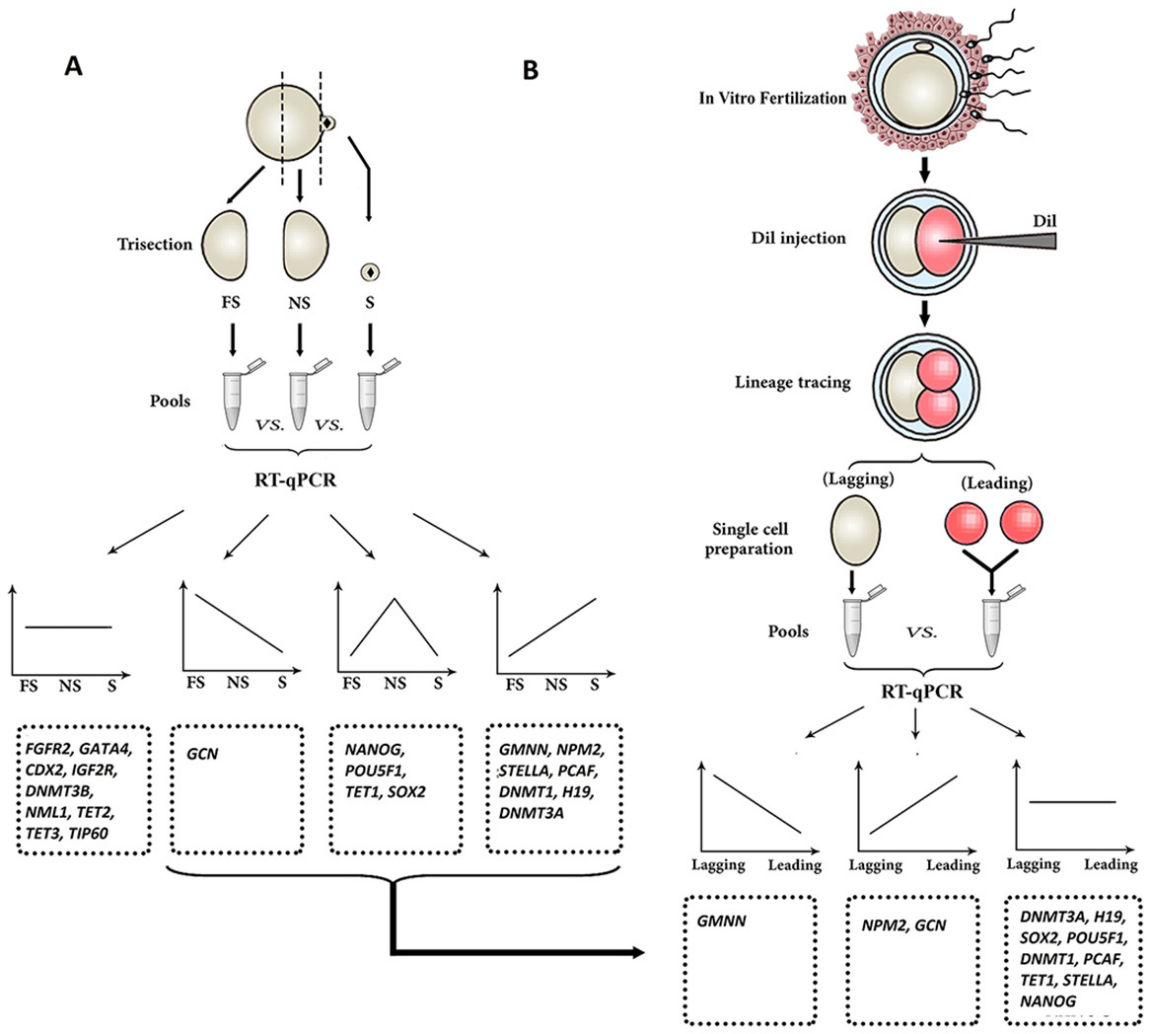
Investigation of transcript assymetry within oocyte and early embryo. A) Unfertilized ovine MII-oocytes were micro surgically trisected to generate cortical materials containing MII-spindle material (S) and oocyte hemispheres that were either near to (NS) or far from (FS) the MII-spindle. The pools of S, NS, and FS cytoplasmic fragments were prepared and used for comparative RT-qPCR using 21 developmentally important genes. Obtained profiles revealed four patterns of transcript abundances within the three fragments of the oocyte. B) To understand whether ovine early embryos inherit differential transcripts localization observed within MII-oocyte, the transcriptional distribution of transcripts between early embryonic sister blastomeres was investigated using RT-qPCR. For this purpose, one blastomere of ovine early embryo was labeled with Dil and the embryos were followed during the first and second embryonic divisions to generate pools of leading and lagging blastomeres. The leading and lagging pool of blastomeres were used for quantitative analysis of those transcripts that were found to be differentially located with unfertilized MII oocytes.

To understand the spatial distribution of transcripts within unfertilized MII-oocytes, a method of oocyte trisection was used to generate cortical materials containing MII-spindle material (S) and oocyte halves that were either near-to (NS) or far-from (FS) the MII-spindle (Figs 1 and 2A, S1 Video). For this purpose, MII-oocytes were first treated with pronase (0.05% in HTCM199) to remove the zona. It has been previously shown that after zona removal, ovine MII-oocytes partially extrude the MII-spindle and associated chromosomes as a clearly visible cytoplasmic protrusion. This cytoplasmic protrusion is easily discernible as a reference position for proper bisection of MII-oocyte to the NS and FS halves as described elsewhere [40]. The obtained NS halves were then used for the preparation of cortical materials containing MII-chromosomes (S) and enucleated NS halves using an inverted fluorescent microscope (Olympus, IX71, Japan) equipped with a micro-manipulator system (Narishige, Japan). Plasma membrane typically encapsulates the isolated MII-chromosome as an intact cytoplasmic fragment. To confirm successful bisection and spindle removal, oocytes were stained with Hoechst 33342 (5 μg/ml, 5 min) before microsurgery. The trisected oocytes were then visualized by brief UV-exposure. For each replicate, the pools of S, NS, and FS cytoplasmic fragments were prepared from 100–150 oocytes.

### Spatial distribution of maternal mRNA between sister blastomeres of early embryos

To understand transcript distribution between early embryonic sister blastomeres (Fig 2B), ovine 2-cell embryos were collected around 18 h post IVF and used for random labeling of one sister blastomere using the fluorescent dye Dil (1,10-dioctadecyl-3,3,30,30-tetramethylindocarbocyanine perchlorate) as described by Park et al. [35]. In brief, Dil was dissolved in corn oil at 2.5 mg/ml at 60°C, allowed to cool and then used immediately. A very sharp beveled micropipette was backfilled with dye and attached to an inverted microscope (Olympus, IX71, Japan) equipped with Narishige micro-manipulator system. Labeling was accomplished by intracytoplasmic injection of the Dil droplet randomly into one two-cell blastomere. Labeled embryos were then cultured in mSOF medium to monitor cleavage and catch 3 cell embryos before the 3 cell → 4 cell division. Three cell embryos were treated with pronase to remove the zona and gently pipetted in phosphate buffer saline (PBS) to separate blastomeres. Using an inverted fluorescent microscope, singled blastomeres were observed under brief UV exposure to collect sister blastomeres of the second cleavage (originated from the leading blastomere) and the lagging blastomere (originated from the first cleavage) in two separate pools (Fig 2). Samples in minimum extraneous media were separately drawn into a pipette and bubbled into RLT buffer to be kept in frozen (-70°C) until RNA extraction and quantitative real-time PCR (RT-qPCR).

### Spatial distribution of crude proteins within ovine unfertilized MII oocytes

To understand the spatial distribution of proteins within unfertilized MII-oocytes, pools of S, NS, and FS oocyte fragments (300 each) were prepared by the manual method of oocyte trisection (Figs 1 and 2A, S1 Video). Oocyte fragments were washed twice by centrifugation in PBS. Fragments were then removed from PBS and lysed by gentle pipetting in 150 μl of TRI reagent (Sigma) according to the manufacturer’s protocol. Protein concentration was measured according to the Bradford method (Bio-Rad) according to the manufacturer’s recommendations. In brief, a solubilized protein fraction of each sample (10 μg) was subjected for sodium dodecyl sulfate (SDS)-12% polyacrylamide gel (PAGE) electrophoresis. The gel-fixing step (30% ethanol, 10% acetic acid) was performed for 4 h and then the gel was stained with commercial silver stain. The SDS-PAGE was continuously developed for 10 min to reveal protein spots. As molecular weight standards, protein molecular weight markers (14–200 KD, Amersham) were run simultaneously.

### Spatial distribution of specific proteins within ovine unfertilized MII oocytes

To understand polarized distribution of special maternal proteins within unfertilized MII-oocytes, a total of 3752 ovine MII oocytes from 15 replicates were used for manual bisection to prepare enough NS and FS oocyte fragments for immunoblotting (Figs 1 and 2A, S1 Video). To ensure the specificity of antibodies, immunoblotting was performed on ovine tissues (testis and liver) and fibroblasts. The procedure of immunoblotting was as previously described [31]. In brief, protein extraction was carried out with TRI reagent (Sigma-Aldrich). SDS-PAGE was performed at 120 V for 2 h using a Mini-PROTEAN Tetra cell (Bio-Rad). Separated proteins were transferred to a polyvinylidenedifluoride (PVDF; Bio-Rad) membrane by wet blotting) Bio-Rad). Membranes were blocked for 1 h with 10% skim milk and incubated for 1.5 h at RT with the respective primary antibodies: anti-NANOG (Sigma#N3038, 1:200), anti-DNM3A (IMG#268A, 1:1000) and anti-glyceraldehyde phosphate dehydrogenase (anti-GAPDH) (Sigma-Aldrich#A2228, 1:5000). At the end of the incubation period, membranes were rinsed with a mixture of Tris-buffered saline with tween 20 (TBST, 0.1% V/V) three times for 15 min each, followed by incubation with HRP-conjugated secondary antibody (goat anti-mouse (Dako#P0447, 1:5000)). HRP-conjugated IgG binding to the protein bands was visualized by an Amersham ECL Advance Western Blotting Detection Kit (GE Healthcare).

### Topological assessment of SEP in ovine eggs

To investigate whether SEP is random or specified in ovine eggs, we examined the topological location of SEP in fertilized oocytes. In pilot experiments to determine the optimal time window for achieving highest early fertilization, ovine eggs at different intervals (h) post-fertilization (hpf: 3, 4, 5, 6, 7, 8, and 12) were denuded of surrounding cumulus cells and then treated with 0.25% pronase to remove the zona. Zygotes were then fixed, stained with Hoechst-33342 and scored for the presence of condensed sperm head at the oocyte periphery as described by Schurmann et al. [41]. In another set of pilot experiments to determine the relationship between MII-spindle and the first polar body (pb), unfertilized oocytes were fixed in 4% paraformaldehyde (PFA) in PBS and stained with Hoechst-33342. Then, the topological relationship between MII spindle and pb was assessed by using a fluorescence-equipped micromanipulation microscope as described by Rienzi et al. [42]. The first set of pilot experiments established that an interval of 4–6 hpf is optimal for achieving maximum early fertilization with the condensed peripheral sperm (S1 Table). Obtained results of the second set of pilot experiment (S2 Table) established that in agreement with the studies of Hardarson et al. [43] and Rienzi et al. [42] in human, pb is a weak indicator of the MII-spindle because it greatly displaces and deviates in spatial position relative to MII-spindle, particularly after mechanical denudation by vortexing.

The procedure of SEP assessment was according to Motosugi et al. [18] with some modifications. In brief, the cumulus-corona was dispersed 4–6 hpf (equals 26–28 h post-IVM) by gentle pipetting in 300 IU/ml hyalorunidase in HTCM199, stained with Hoechst-33342 and transferred to the stage of micromanipulator microscope. A small drop of Dil was injected into the zona pelucida directly overlying meiotic spindle using a fluorescent-equipped micromanipulator (Olympus, BX51, Japan). Only zygotes having a condensed sperm were used for Dil-labeling of meiotic spindle position and unfertilized oocytes or polyspermic zygotes were excluded. Labeled zygotes were incubated in 0.5% sodium citrate dihydrate for a few minutes until part of the cytoplasm protruded through a tiny hole in the ZP that was introduced by the penetrating sperm. Presumptive zygotes with a clear protrusion were fixed in 4% PFA in PBS, stained with Hoechst-33342 and evaluated for the SEP using a fluorescent-equipped micromanipulator (Olympus, BX51, Japan). To map SEP, zygote were rotated under constant UV-light and using the micromanipulator to align the two points of interest, the meiotic spindle (labeled by Dil injection into overlying ZP) and the site of sperm penetration (based on the site of cytoplasmic protrusion from ZP) in the focal plane traversing the equatorial plane. To determine the spatial relationship between SEP and meiotic spindle, manipulated zygotes were subdivided into four imaginary zones (zones I-IV), representing 3 × 30° discs and 1×180° disc. In this scheme, zone-I was the closest to the meiotic spindle, whereas zone-IV was the furthest from the meiotic spindle.

### Topological relationship between SEP and the first cleavage plane in ovine early embryos

To understand possible topological relationship between SEP and the first cleavage plane, presumptive zygotes at 4–6 hpf were stripped of cumulus cell, stained with Hoechst-33342 and transferred to the stage of micromanipulator microscope. To map the SEP, eggs were rotated using micromanipulator and under constant UV light until the point of interest, the condensed penetrated sperm, was adjusted at 3 O’clock position. A single oil drop was injected into the zona directly overlying the SEP. Labeled zygotes were returned backed into their embryo culture condition to monitor cleavage and early 2 cell embryos around 8–12 h post zona marking. For documentation and analysis, 2-cell embryos were fixed and then rotated under constant UV-light and using the micromanipulator to align the two points of interest, the SEP (labeled by Dil injection into overlying ZP) and the cleavage plane, in the focal plane traversing the equatorial plane. The spatial relationship between SEP and cleavage plane was measured as described above.

### Spatial distribution of lipid droplets between sister blastomeres of ovine early embryos

The lipid content of 2-cell embryos has been linked to the biased embryonal axis formation in porcine [34]. To investigate this possibility in the ovine, 2-cell embryos were used for the analysis of total lipid content using Nile red, a fluorescent dye specific for intracellular lipid droplets [34, 44]. After thorough washing in PBS, samples were incubated with 0.2 μg/ml of Nile red overnight at 4°C. Samples were then counterstained with Hoechst-33342, washed in PBS and mounted on glass microscope slides in one drop of antifading. After gentle compression with a coverslip, samples were visualized using an epifluorescence microscope (Olympus, BX51, Japan). Upon exposure, a digital image of each sample was taken with a high sensitive camera (Olympus DP-72) operated on DP2-BSW Software. Using Image J. software (National Institute of Mental Health, Bethesda, Maryland, USA), the mean fluorescent intensity between the blastomeres of two-cell stage embryos was analyzed.

### Fluorescent labeling of the blastomeres

One blastomere of two-cell stage ovine embryos was randomly labelled with fluorescent Dil. After preparation of injection needle as described above, random labeling of one two-cell blastomere was accomplished by intracytoplasmic injection of the Dil droplet as described by Park et al. [35]. Labeled embryos were subsequently cultured in their respective embryo culture media and checked for the second cleavage every 3–4 h under a stereomicroscope. Three-cell stage embryos were subsequently inspected with an inverted fluorescent microscope for the sequence of the cleavage. Embryos were classified into the following two groups: 1) leading embryos in which the labeled blastomere cleaved first, 2) lagging embryos in which the unlabeled blastomere cleaved first. Arrested embryos (none of the two-cell blastomeres cleaved during observation) or embryos with lysed injected blastomere were discarded from the experiments and intact leading and lagging embryos were cultured for further 120 h in mSOF. To understand if Dil lineage-tracing may delay embryo development, the frequency an order of cleavage in the labeled and non-labeled blastomeres of the two-cell stage embryos were recorded.

### Observation of the embryos

**Fig 3:**
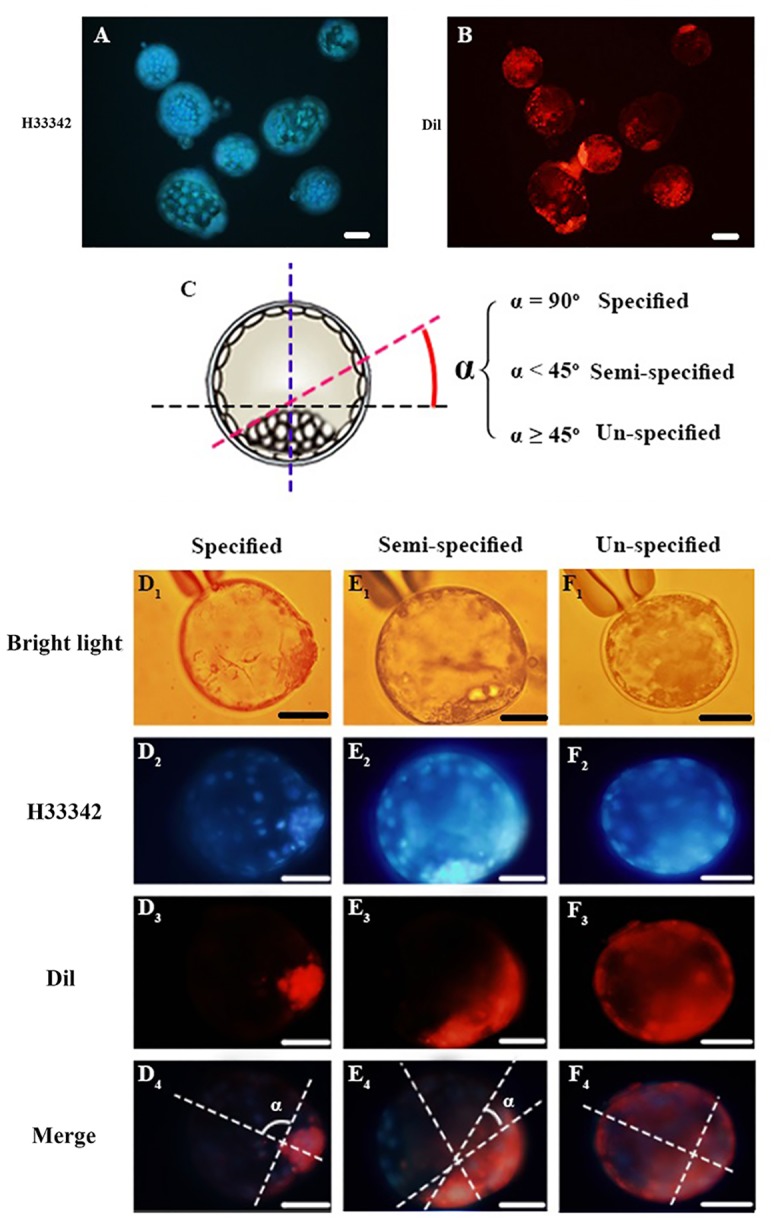
Lineage tracing of the leading and lagging blastomeres. A cohort group of blastocysts derived from leading-labeled two-cell embryos that are observed using appropriate Hoechst-33342 (A) and Rhodamine (B) filters. C) Criteria for sorting of Dil-labeled embryos. Scoring the orientation of the Em-Ab axis in ovine blastocysts relative to the first cleavage plane of the 2-cell embryo. After drawing imaginary lines of the Em-Ab axis and equatorial plane, the blastocysts were classified to three groups depending on the angular departure of the boundary line of the fluorescent/nonfluorescent cells from the equatorial plane: i) specified: when the angular departure was almost 90°, ii) semi-specified: when the angular departure was ≥45°, iii) un-specified: when the angular departure was <45°. D1-F4) Representative images of specified (D1-D4), semi-specified (E1-E4) and un-specified (F1-F4) observed under bright light (D1, E1, & F1), and H33342 (D2, E2, & F2) and Rhodamine (D3, E3, & F3) filters. D4, E4, & F4 images show merged images of H33342 and Rhodamine. Bar represents 100 μm.

In mice blastocysts, there is no agreement regarding the angular degree between the Em-Ab axis and the first cleavage plane of the two-cell embryo is controversial [8, 12, 45]. Therefore, the first cleavage plane is defined by a best-fit boundary between the coherent fluorescent and non-fluorescent cells. Based on the angle between he first cleavage plane and Em-Ab axis (≤30° or >30°), the labeled embryos were categorized as orthogonal or deviant [12]. We used a modified method of blastocyst scoring by a best-fit approach to ovine blastocysts (Fig 3).

In brief, embryos at the blastocyst stage were fixed, stained with Hoechst-33342 and used for imaging using an inverted microscope (Olympus, IX71, Japan) equipped with the appropriate Rhodamine and Hoechst-33342 filters. Under bright light, each blastocyst was initially held with a holding pipette to position ICM at either 3 or 9 O’clock. In this scheme, the equatorial plane separating the entire ICM and polar TE from the remaining part of TE ran parallel to the plane of view and the Em-Ab axis of the blastocyst ran perpendicular to the equatorial plane [12]. With the aid of a glass needle attached to a Narishige micromanipulator system, the blastocyst was then carefully rotated along the embryonic-abembryonic axis to determine the number of cells derived from the leading and lagging that occupying the Em and Ab parts. The obtained serial of images was used for making a whole view of localization of colored and non-colored cells with the blastocyst. Using these images, the blastocysts were classified to three groups depending on the angular departure of the boundary line of the fluorescent/nonfluorescent cells from the equatorial plane: i) specified: when the angular departure was almost 90°, ii) semi-specified: when the angular departure was still <45°, iii) un-specified: when the angular departure was ≥45°.

### Developmental competence of the corresponding sister blastomeres

To understand the developmental competence of sister blastomeres in ovine embryos, 2-cell stage embryos were used for separation and in vitro culture of the sister blastomeres as described by Held et al. (2012) [46]. In brief, zona-pelucida of 2-cell embryos was removed by brief treatment with 0.25% pronase that was prepared in hepes-buffered SOF (HSOF) for 1–2 min. Sister blastomeres were separated by gentle pipetting in Hepes-SOF. Zona-free separated blastomeres were cultured individually in micro-wells. In brief, 20 μl droplet of mSOF were prepared in the bottom of a non-adhesive 35 mm Grainer dish under mineral oil. Using a sterilized home-made borer, 12–16 small wells were bored in two parallel rows in the plastic bottom of each culture droplet. After equilibration, the corresponding sister blastomeres derived from 2-cell stage embryos were placed individually in parallel micro-wells. These blastomeres were cultured for 8 further days at 39°C, 5% O_2_, 6% CO_2_, and maximum humidity. Subsequent cleavage and development of the sister blastomeres to the blastocyst stage were evaluated at Days 3 and 8, respectively. Developmental competence of the corresponding sister-blastomeres was classified as: 1) one further cleavage (2-cell block: 2CB), 2) two further cleavage (4-cell block: 4CB), 3) three further cleavage (8-cell block: 8CB), 4) four further cleavage (morula block: MB), and those blastomeres that reached the blastocyst stage (BLS). Those sister blastomeres that were stopped without any further cleavage after separation were few and were not included in this experiment.

### Quantitative real time PCR (RT-qPCR)

The transcript abundances of 21 genes (Table 1) were compared between pools of trisected MII oocyte fragments; S, NS, and FS. To select the genes, we sought RNA expression databases to find genes that having one gene ontology designation related with pluripotency and differentiation (*SOX2*, *NANOG*, *POU5F1*, *FGFR2*, *GATA4*, *CDX2*), Nuclear remodeling (*NPM2* and *GMNN*), imprinting (*H19* and *IGF2R*), and epigenetic modification of DNA or histone (*PCAF*, *DNMT3*A, *DNMT3*A, *DNMT1*, *TET1*, *TET2*, *TET3*, *KAT5*, *MLL1*, *PCAF*, and *STELLA*). The procedure for RT-qPCR was as described previously [47]. In brief, total RNA was extracted using RNeasy Micro kit (Qiagen, Mississauga, ON, Canada) followed by the treatment with DNase-I (Ambion, Streetsville, ON, Canada) according to the manufacturer’s protocol. The RNA quality and quantity was determined using the WPA Biowave spectrophotometer (Cambridge, United Kingdom). For reverse transcription, 10 μl of total RNA was used in a final volume of 20 ml reaction containing 1 μl of Random Hexamer, 4 μl RT buffer (10X), 2 μl of dNTP, 1 μl of RNase inhibitor (20 IU), and 1 μl of reverse transcriptase (Fermentas, Glen Burnie, Ontario, Canada). Reverse transcription was carried out at 25°C for 10 min, 42°C for 1 h and 70°C for 10 min. The master mix was prepared using 1 μl of cDNA (50 ng), 5 μl of the SYBR Green/0.2 μl ROX qPCR Master Mix (2X) (Fermentas, Germany) and 1 μl of forward and reverse primers (5 pM) adjusted to a total volume of 10 μl using water nuclease-free. Three technical replicates of RT-qPCR were conducted for each primer. CT samples of each target gene were normalized to the CT of *GAPDH* (because of its stable expression between groups) and represented as 2^-ΔΔCT^ [48].The primer sequences, annealing temperatures and the size of amplified products are shown in Table 1.

**Table 1:** Specific primers used for qRT-PCR.

Gene Symbol	Description	Function*	Sequence (5`to 3`)^F^_R_	Tm (°C)	Length
***H19***	H19	Imprinted maternally expressed transcript (non-protein coding	CCTAAAGGAACGGAAGAC RGGAGGCTGTTGGTGGT	58	100
***PCAF***	K (Lysine) Acetyltransferase 2B	Histone acetyltransferase and promotes transcriptional activation.	ACGAACAAGTCAAGGGCTATG CAGAGAACTCCGTGTATGGG	58	97
***DNMT3A***	DNA (Cytosine-5-)-Methyltransferase 3 Alpha	Abundantly expressed in ES cells.	AAAGCCCCGAAAGAGC AGAGGGTGTTCCAAGGTG	59	107
***DNMT1***	DNA (Cytosine-5-)-Methyltransferase 1	Constitutively expressed in several tissues. An important mediator of the methylation process	GAAGCAGAACAAGAATCGG TTTGAAGAGTCGTCTGGAA	57	99
***STELLA***	Developmental Pluripotency Associated 3	Primordial germ cell (PGCs)-specific protein. Protects maternal genome from TET3-mediated conversion to 5hmC.	ATAGGACTACGCCCATTC TGTAGGCTCAAACTGCTC	58	97
***SOX2***	SRY (Sex Determining Region Y)-Box 2	Critical for early embryogenesis and for embryonic stem cell pluripotency.	GCCGCCGATGATTGTTAT AGAGAGAAAGAAAGGGAGAGAA	56	78
***TET1***	Tet Methylcytosine Dioxygenase 1	Involved in the balance between pluripotency and lineage commitment of cells and in inner cell mass cell specification.	CAAGAGCCAAGAGATAACC GTCCTTCTCCACCATACG	57	103
***GCN2***	Eukaryotic Translation Initiation Factor 2 Alpha Kinase 4	Histone acetyltransferase.	ACTCACCTGATGAACCAC TGTCGCACCCTCGTAG	52	107
***FGFR2***	Fibroblast Growth Factor Receptor 2	Cell proliferation, differentiation and migration	GTGATGTCTGGTCCTTCG GAAGGTGGGTCTCTGTGA	55	105
***CDX2***	Caudal Type Homeobox 2.	Trophectoderm differentiation	CCCCAAGTGAAAACCAG TGAGAGCCCCAGTGTG	56	107
***TIP60***	K(Lysine) Acetyltransferase 5	Transcriptional activation of select genes principally by acetylation of nucleosomal histones H4 and H2A.	AAACGGAAGGTGGAGGT ATGCGAGGAGCAGACG	56	105
***DNMT3B***	DNA (Cytosine-5-)-Methyltransferase 3 Beta	Essential for de novo DNA methylation and development (at least in mouse)	GGAAGGAGTTTGGAATAGG CTTATCTGCTGGAATCTCG	54	104
***IGF2R***	Insulin-Like Growth Factor 2 Receptor	Cell lineage specification.	CCAGAACCTGACCCTC CTCTGCCGTTGTTACCT	58	103
***GATA4***	GATA Binding Protein 4	Transcription factor. Involved in lineage determination.	TCCCCTTCGGGCTCAGTGC GTTGCCAGGTAGCGAGTTTGC	63	108
***TET2***	Tet Methylcytosine Dioxygenase 2	Active DNA demethylation and epigenetic reprogramming.	ACTGTGTGCCCCGAGAT TAGTTGTGTTTGTGCTGCC	61	104
***TET3***	Tet Methylcytosine Dioxygenase 3	Paternal pronucleus DNA demethylation.	CATCAAGCAAGAGCCAGTAG TCCACAGGAGGGAACACG	62	109
***MML1***	Lysine (K)-Specific Methyltransferase 2A	Acts in association with HOXA9 for anterior-posterior axis positioning.	CTGACAAGAGTGTGGAGAAG CTACCCATAGGATACAAAGC	63	99
***GAPDH***	Glyceraldehyde-3-Phosphate dehydrogenase	Reference gene	GTTCAACGGCACAGTCAAG TACTCAGCACCAGCATCAC	60	117

*: Gene functions were adopted from GeneCards^®^ human gene database.

### RNA integrity analysis

To assess the integrity of total RNA, aliquots of the RNA samples were run on a denaturing agarose gel stained with ethidium bromide. In this scheme, intact total RNA run on a denaturing gel had sharp and clear 28S and 18S rRNA bands.

### Statistical analysis

All experiments were replicated at least three times. Before any statistical analysis, the normality of data was evaluated. Percentages data were transformed by ArcSin and analyzed by one-way ANOVA model of SPSS version 17 (SPSS, Science, Chicago, IL, USA). Differences were compared by the Tukey multiple comparison post hoc test. All data are expressed as mean ± S.E.M. and differences were considered as significant at P<0.05.

## Results

### Fertilizing sperm enter preferentially through the meiotic spindle half in ovine oocytes

**Fig 4:**
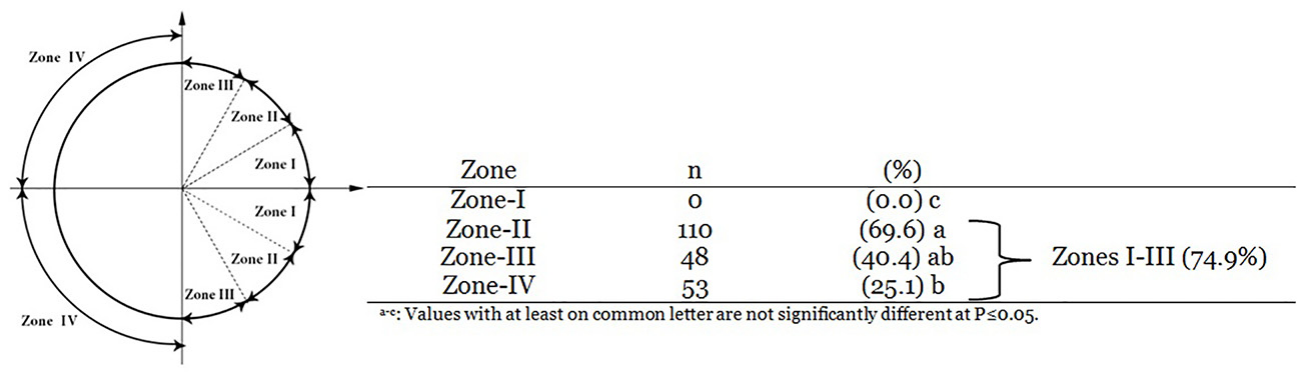
Topological assessment of sperm entry point (SEP) in ovine eggs. Fertilzied oocytes were rotated under constant UV-light until MII-spindle was positioned to 3 O’clock. Then, the spatial relationship between SEP and MII-spindle was measured in 4 imaginary oocyte zones. In this scheme, zones I and IV are the closest and the furthest from the MII-spindle, respectively. Table represents the results of topological assessment of SEP.

The topological relationship between meiotic spindle and SEP is summarized in Fig 4. A total of 211 monospermic zygotes were used for SEP assessment in three replicates. The percentages of zygotes showing SEP in the meiotic and non-meiotic halves were 74.9% (n = 158) and 25.1% (n = 53), respectively. Importantly, the distribution of SEP in the meiotic half was not random as any (0%) SEP was observed in zone-I (~16.8% of total oocyte area that is the closest to the meiotic spindle). The proportion of SEP at zone-II (the second ~16.8% of total oocyte area that is the closest to the meiotic spindle) was significantly higher than zone-III (69.6 vs. 40.4%, respectively).

### Some maternal mRNAs are asymmetrically distributed within the MII-oocyte in ovine

**Fig 5:**
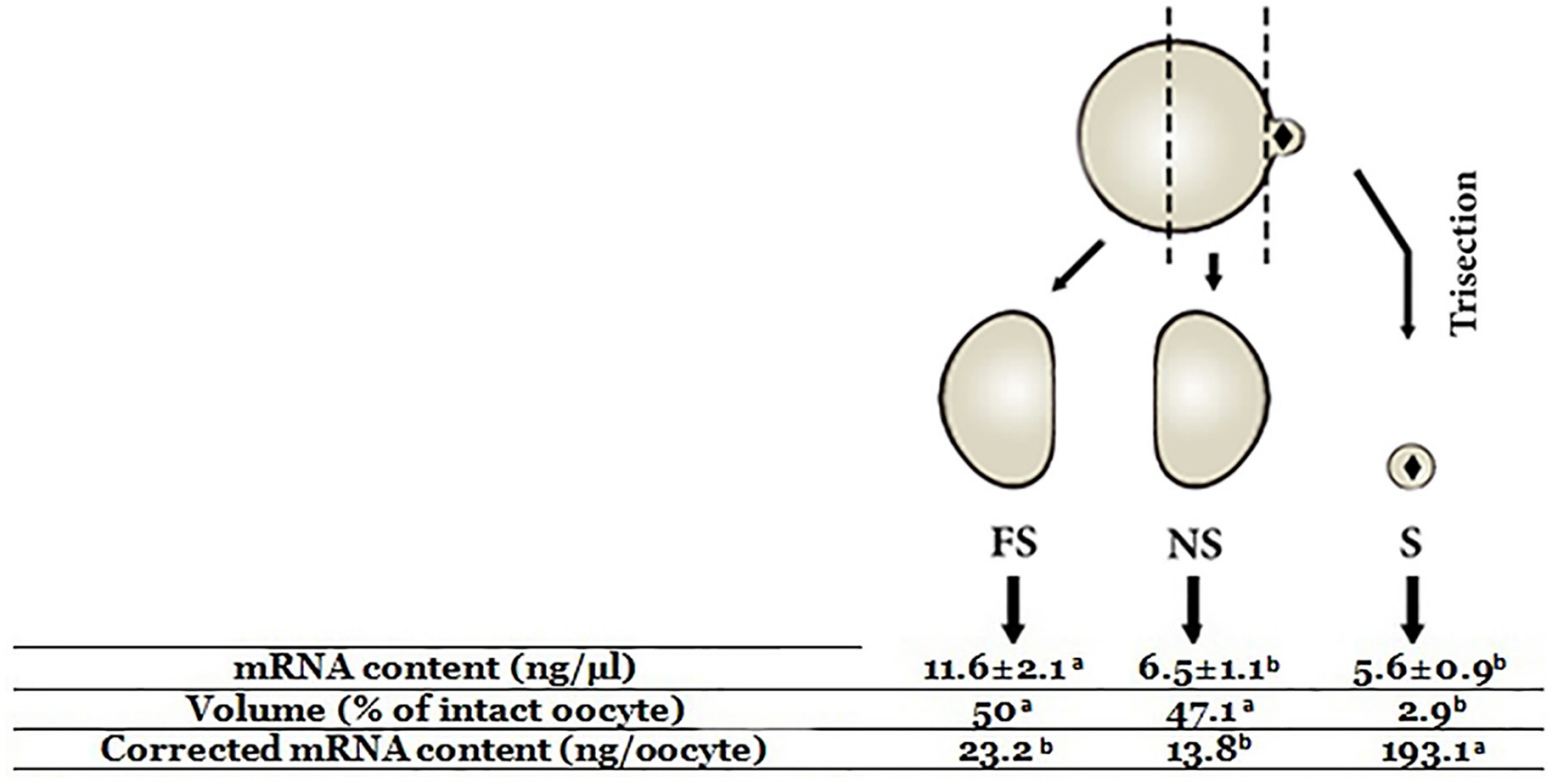
Comparative analysis of total mRNA contents between subcellular structures of MII-oocyte. ^a-c^: values with different superscripts are significantly different at p<0.05.

The spatial gradient of maternal mRNA was compared between pooled S, NS, FS oocyte fragments that were prepared by microsurgical trisection of 450 ovine unfertilized MII oocytes in three replicates. As shown in Fig 5, the total mRNA content of the pooled FS-substructures (11.6±2.1 ng/μl) was comparable to the cumulative mRNA content of NS (6.5±1.1 ng/μl) + S (5.6±0.9 ng/μl) -substructures. The total mRNA content of S oocyte fragment was significantly lower than FS but NS counterparts. Even though, since the isolated S oocyte fragment comprise only ~2.9% of total oocyte volume, the proportional mRNA content of S-substructures was estimated to be 8 and 14 fold higher than NS and FS oocyte fragments, respectively.

**Fig 6:**
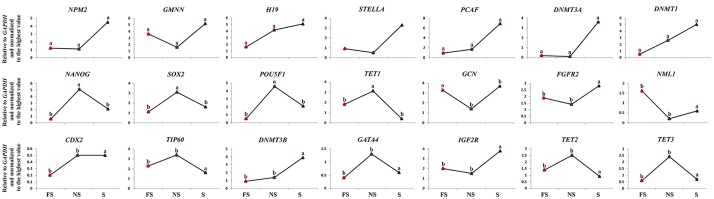
Expression of selected genes in subcellular structures of MII-oocyte. ^a-c^: values with different superscripts are significantly different at p<0.05.

Quantitative comparison of transcripts between S, NS, and FS oocyte fragments (Fig 6) revealed that of 21 transcripts analyzed, 12 (57%) and 9 (43%) showed asymmetric and symmetric distributions within the MII-oocyte, respectively (Figs 2A and 6). Based on their patterns of regionalization within MII-oocyte, these transcripts can be categorized to those transcripts that were i) more abundant in S compared to either NS or FS counterparts (*NPM2*, *GMNN*, *H19*, *PCAF*, *DNMT3a*, *DNMT1*, and *STELLA*), ii) more abundant in NS compared to either S or FS counterparts (*SOX2*, *NANOG*, *POU5F1*, and *TET1*), iii) more abundant in FS compared to either NS or S counterparts (*GCN*), and iv) evenly distributed throughout the oocyte (*FGFR2*, *NML1*, *CDX2*, *TIP60*, *DNMT3B*, *IGF2R*, *GATA4*, *TET2* and *TET3*).

To assess the integrity of total RNA, an aliquot of each RNA sample was run on a denaturing agarose gel stained with ethidium bromide. Intact total RNA run on a denaturing gel will have sharp, clear 28S and 18S rRNA bands, indicating the RNA quality (S1 Fig).

### Sister blastomeres of 3-cell embryos do not inherit the asymmetric transcript abundances of MII-oocyte in ovine

**Fig 7:**
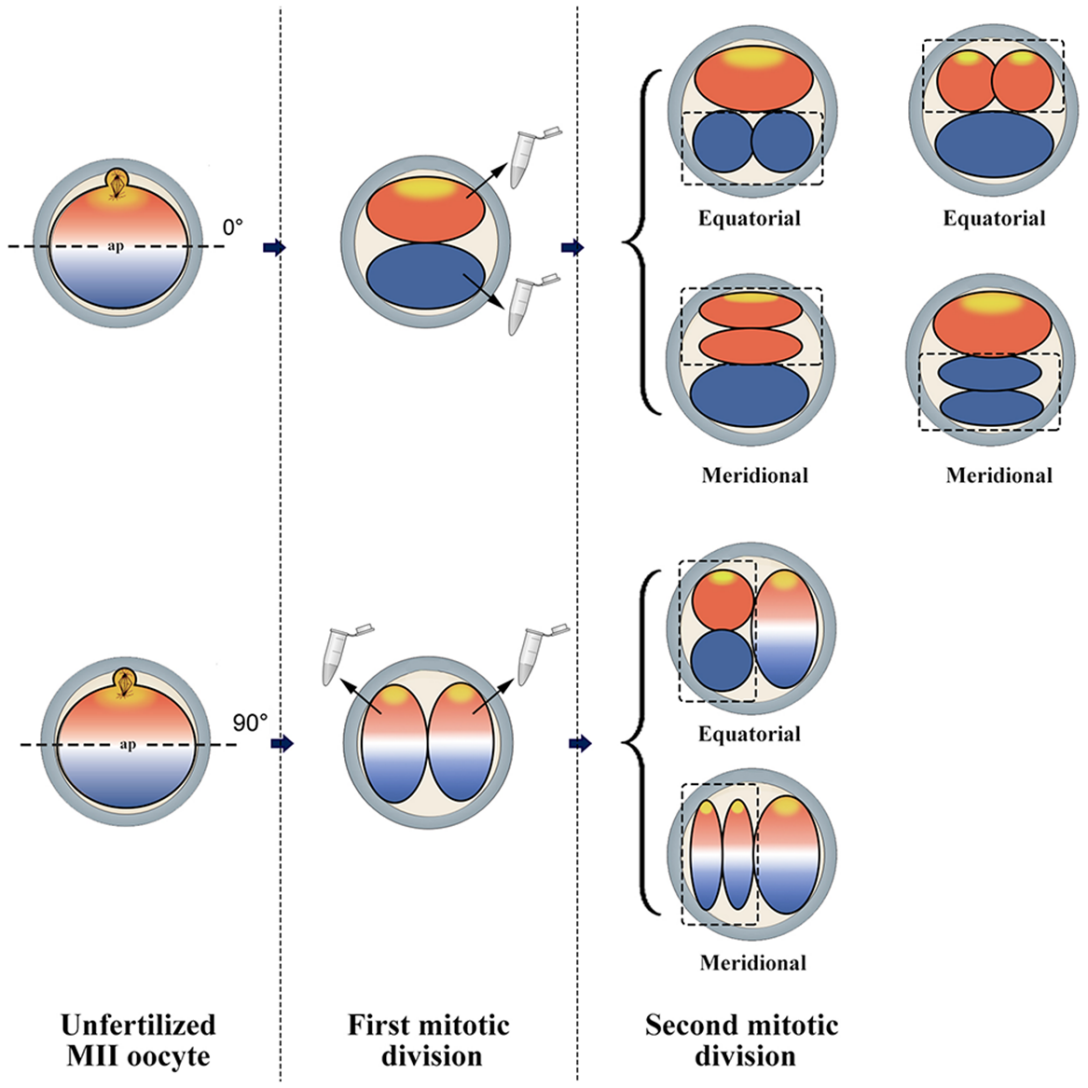
The importance of orientation of the embryonic divisions. Cleavage can occur either equatorially or meridionally, along the animal-vegetal axis, with reference to MII-spindle as the hypothetical animal pole. A result of equatorial first cleavage division is the transcriptome asymmetry between the balstomeres of 2-cell embryo that could persist even after the second cleavage division. By contrast, a result of meridional first cleavage division is the transcriptome symmetry between the balstomeres of 2-cell and 3-cells embryos, despite oocyte transcript polarity. Moreover, indiscriminate separation of 2-cell embryos yields constraints that exclude the possibility that transcriptome asymmetry within MII-oocyte leads to programmed asymmetric transcriptome inheritance between blastomeres of 2-cell embryos.

Since 12 transcripts showed asymmetric regionalization within MII-oocyte, we wished to know whether the same pattern of asymmetry can be inherited by the resulting embryos. Given that one blastomere of 2-cell embryos cleaves first, indiscriminate separation of 2-cell embryos yields constraints that exclude the possibility that transcriptome asymmetry within MII-oocyte leads to programmed asymmetric transcriptome inheritance between blastomeres of 2-cell embryos [4] (Please see Fig 7). To avoid this unwanted bias, RT-qPCR was performed between sister blastomeres of the second cleavage and the remaining blastomere of the first cleavage (Fig 2B). Of 12 transcripts evaluated, 9 (75%) transcripts (*H*19, *PCAF*, *DNMT3A*, *DNMT1*, *STELLA*, *SOX2*, *NANOG*, *POU5F1*, and *TET1*) showed comparable abundances between the second mitotic products, suggesting no evidence of programmed inheritance in ovine early embryos. Even though, 3 of 12 transcripts (25%) were asymmetrically distributed between the sister blastomeres of 3-cell embryos. The *NPM2* and *GCN* transcripts were significantly higher in leading vs. lagging blastomere, whereas *GMNN* was significantly higher in lagging vs. leading blastomere.

### Total maternal proteins are asymmetrically distributed within the MII-oocyte in ovine

**Fig 8:**
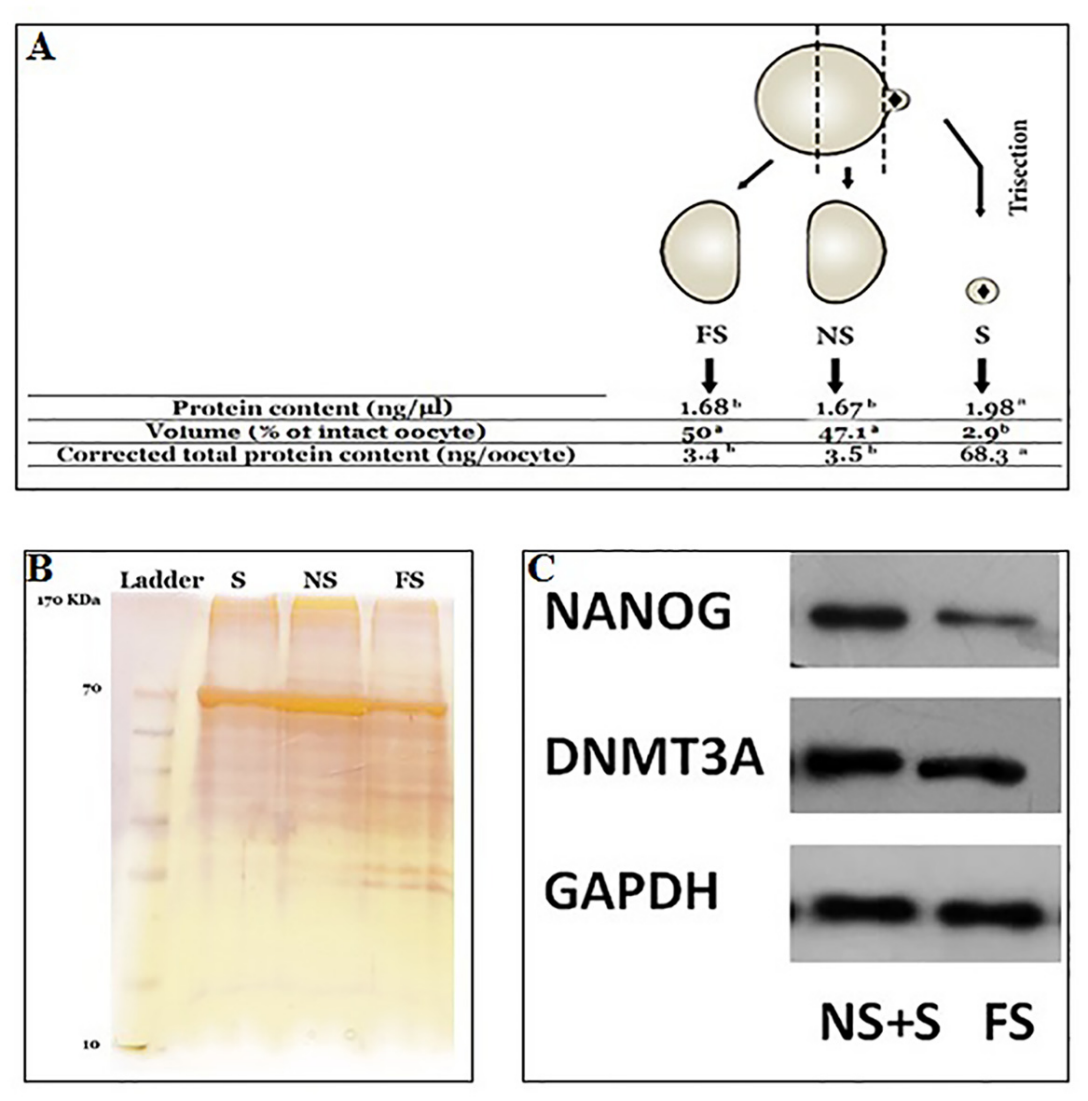
A) Comparative analysis of total protein contents within subcellular structures of MII-oocyte. ^a-c^: values with different superscripts are significantly different at p<0.05. B) Protein patterns of oocyte substructures revealed by SDS-PAGE electrophoresis. C) Western blot analysis of NANOG and DNMT3A between NS+S and FS oocyte fragments.

The spectrophotometry analysis of protein gradients (Fig 8A) showed that total protein contents of S, NS and FS oocyte fragments were very similar (1.98, 1.67 and 1.68 ng/μl, respectively). Even though, total protein content of S+NS substructures (3.65 ng/μl) was two-fold higher than FS. Moreover, considering the fact that S oocyte fragment comprises only around 2.9% of total oocyte volume, the proportional protein content of S oocyte fragment could be estimated about 68.3 ng/μl, which is highly significantly higher the related protein contents of NS and FS counterparts (3.5 and 3.4 ng/μl, respectively).

Total protein lysates of oocyte substructures were electrophoretically separated by molecular weight through SDS-12% PAGE and proteins were visualized by silver staining (Fig 8B). The absence of peripheral smear on the basic edge of the gel indicated that the majority of oocyte proteins that were stainable were resolved during SDS-page electrophoresis. Notably, despite close similarities in the protein patterns of the three oocyte substructures, some quantitative or qualitative differences were observed in the protein patterns on SDS-gel. For example, the intensities of 170 and 70 KDa protein bands in NS were higher than S and FS.

### Specific maternal proteins are asymmetrically distributed within the MII-oocytes in ovine

To ensure the specificity of antibodies, immunoblotting was performed on ovine tissues (testis and liver) and fibroblasts. Obtained data indicated that among seven antibodies (SOX2, DNMT3A, DNMT3B, DNMT1, cKIT, OCT4, and NANOG) that were checked for cross-reactivity with the corresponding ovine proteins, only DNMT3A, DNMT3B and NANOG antibodies reacted with the corresponding ovine proteins. Immunoblotting of bisected ovine oocytes with these approved antibodies indicated that DNMT3A and NANOG proteins were differently localized within the ovine MII oocytes (S2 Fig). Accordingly, the oocyte half near to spindle (NS) encompassed higher levels of maternal DNMT3A and NANOG proteins compared to the oocyte half that was located far from the spindle (FS). While DNMT3B properly reacted with ovine tissues (testis and liver) and fibroblasts, no protein band was obtained with either bisected or intact oocytes (Fig 8C).

### SEP may not determine the first cleavage plane in ovine embryos

To determine whether the SEP has any relationship with the first cleavage plane, SEP was marked using dil-labeling of the zona. Among 68 cleaved eggs analyzed, only in 3 (4.4%) the SEP and cleavage plane overlapped. In the remaining 65 eggs, the topological position of first cleavage plane with regard to SEP was quite stochastic. Using another batch of labeled eggs, we also could not distinguish a tendency for SEP to be located in particular region of that the 3-cells embryos (data not shown).

### Distribution of cytoplasmic lipids are relatively symmetric between ovine early embryonic sister blastomeres

**Fig 9:**
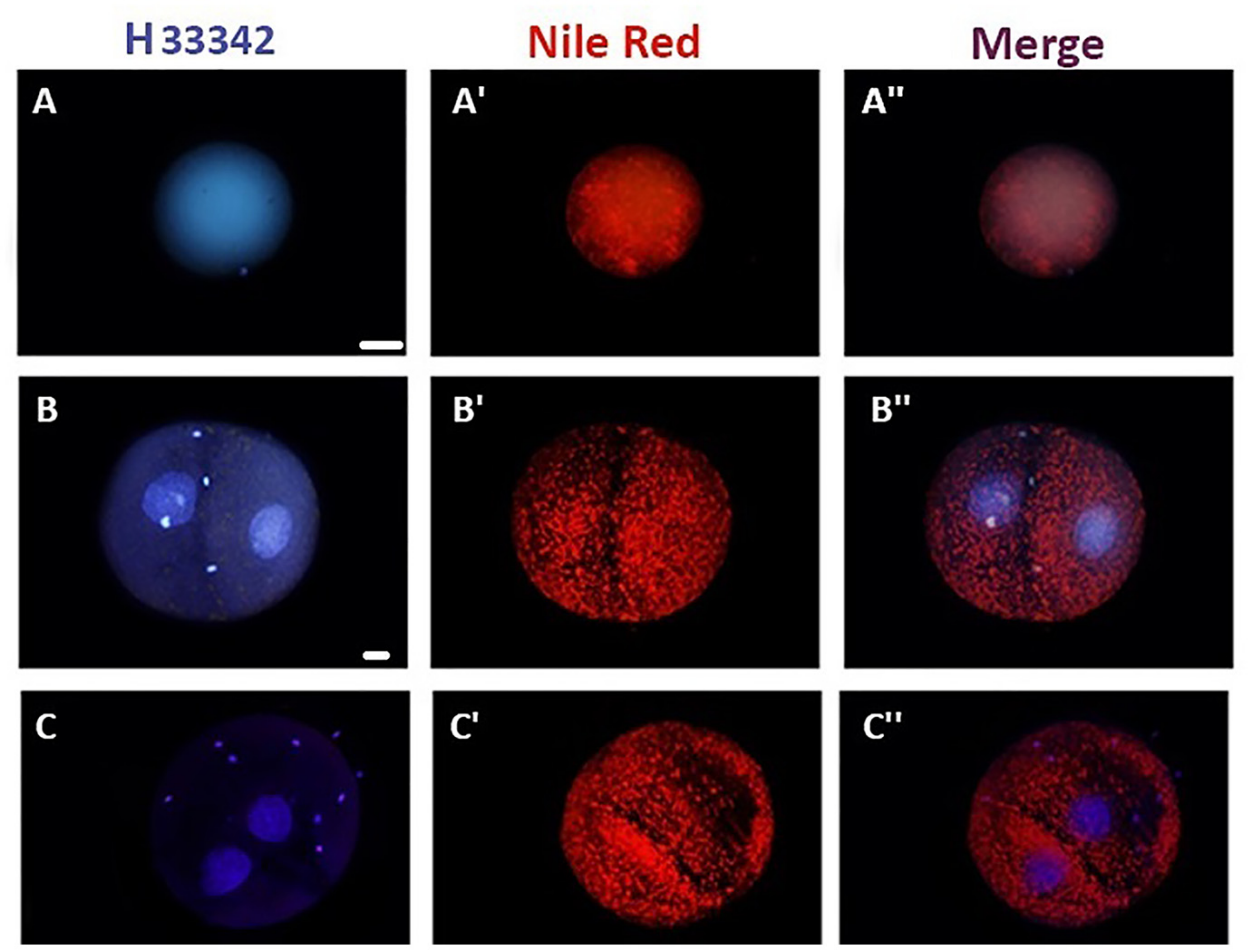
Representative figures and Nile-red staining for investigation of lipid droplet distribution with MII-oocyte (A-A”) and between sister blastomeres of 2-cell embryo (B-C”). Lipid-droplets are evenly distributed within MII-oocyte (A-A”), and in majority of 2-cell embryos (A-A” & D). However, in some 2-cell embryos, one balstomere had significantly higher lipid droplets (C-C” and E). Bar represents 100 μm. *: significantly different at P<0.05.

A lipid-specific fluorochrome, Nile-red, was used for spatial comparison of lipid content within the oocyte (Fig 9, samples images A-A”) and between the blastomeres of 2-cell stage embryos (31 embryos in 3 replicates). (Fig 9, samples images B-C”). One first observation was that the relative fluorescence mean intensity greatly varied between different embryos. Even though, in 40/45 (88.9%) of assessed 2-cell embryos, no significant difference between the relative mean fluorescence intensities of the two blastomeres (0.9±0.2 vs. 1.3±0.3, arbitrary units) (Fig 9, samples images B-B”). In the remaining 5/45 (11.1%) of 2-cell embryos, the relative fluorescence mean intensity of one blastomere was significantly higher than the other one (0.6±0.3 vs. 1.5±0.2 arbitrary units) (Fig 9, samples images C-C”).

### Intracytoplasmic injection of Dil does not compromise cell division and embryo development in ovine

The effect of Dil injection on embryo survival, cell division and embryo development was analyzed. Out of 245 ovine 2-cell embryos that were injected with Dil, 32 (13.1%) were found with lysed blastomeres. Nonetheless, the respective percentage of successfully labeled embryos that could develop to the blastocyst stage (37.5%) was comparable to the sham injection (41.3%) and non-injected control (44.3%). Importantly, the division order of 2-cell blastomeres was independent of the labeling method because the likelihood of labeled and non-labeled blastomeres to divide first was comparable (58.2% (124/213) and 41.8% (89/123), respectively).

### The progeny of leading blastomere contributed to more cells in the developed blastocysts compared to lagging counterpart

**Fig 10:**
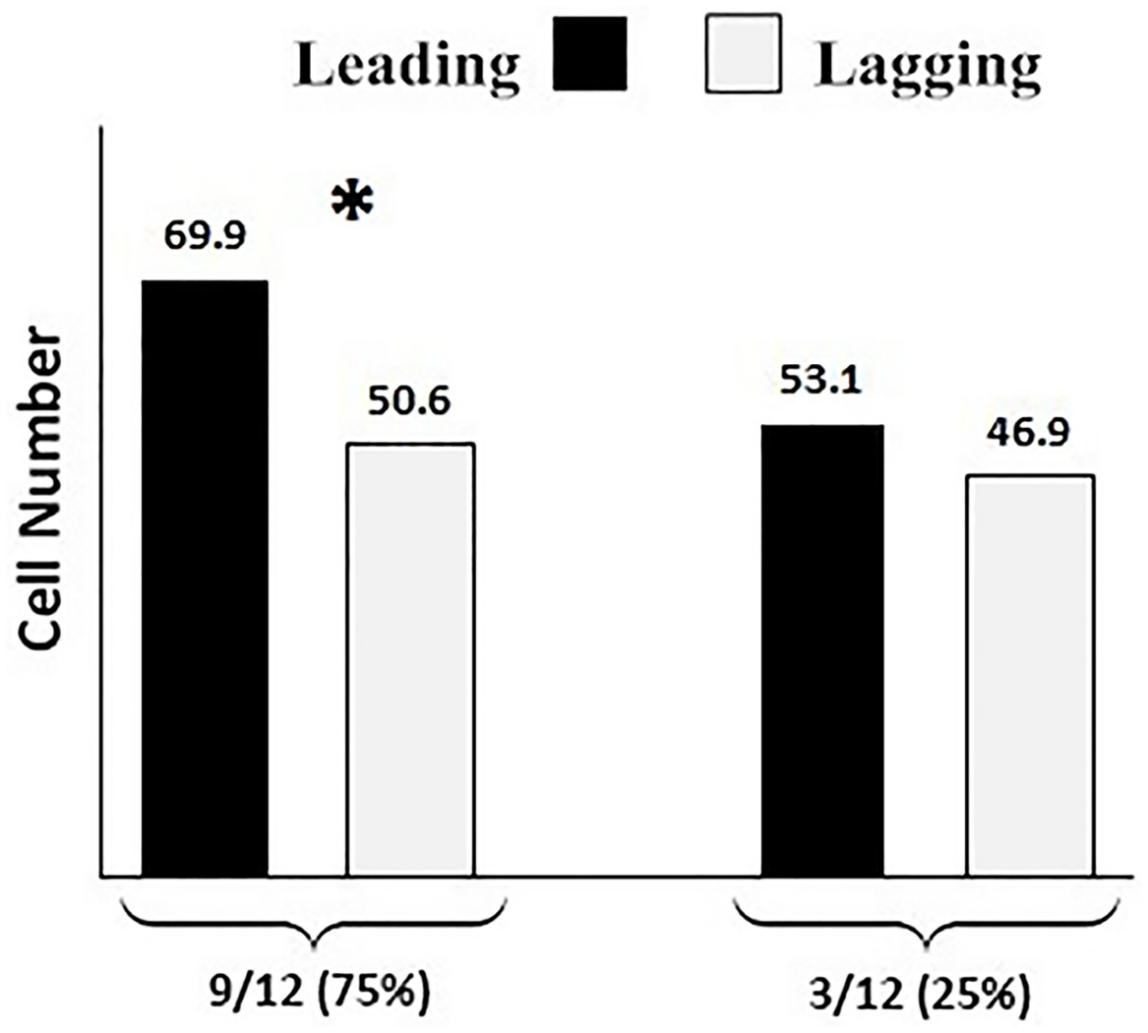
Contributions of leading and lagging blastomeres to the total cell count of the developed blastocysts. In majority (75%) of blastocysts, leading blastomeres contributed to significantly higher cells of the blastocyst compared to the lagging counterpart. In the remaining 25%, the contributions of leading and lagging blastomeres to total cell count of the blastocysts were comparable. *: significantly different at P<0.05.

Of 213 successfully labeled ovine embryos, 80 (37.5%) developed to the blastocyst. In 10 blastocysts (12.5%), we were unable to determine the exact distribution of cells derived from injected blastomere. This failure was due to the vesicular-like (lack of distinguishable ICM) that sometimes occur during in vitro production of embryos in farm animals [49]. The contribution of leading and lagging blastomeres to the total cell number of the developed blastocysts was analyzed in 12 blastocysts (Fig 10). In majority (9/12 = 75.0%), the leading blastomere contributed to more cells in the developed blastocysts compared to lagging blastomeres (on average 69.6 vs. 50.6, respectively). In the remained blastocysts, the overall contribution of leading and lagging blastomeres to the total cell number of the developed blastocysts was comparable (on average 53.1 vs. 46.9, respectively).

### Order of cleavage of two-cell stage ovine embryos does not determine the contribution of leading and lagging blastomeres to the embryonic-abembryonic parts of the developed blastocysts in ovine

Whether leading or lagging blastomere had been labeled in two-cell embryos, only 3.3% of developed blastocysts had an orthogonal angular degree between fluorescent/nonfluorescent cells and the equatorial plane (specified pattern) (Fig 3, Table 2). Moreover, 8.2% of the developed blastocysts represented a mild deviation (<45°) in their angular degree (semi-specified pattern), a ratio that was not significantly different compared to orthogonal (defined) group. However, 88.5% of the developed blastocysts showed ≥45° deviation in their angular degree (un-specified pattern) which was significantly higher than both of the latter groups (Fig 3, Table 2).

**Table 2:** Relationships between the progenies of leading and lagging balstomeres, embryonic axis and equatorial plane.

Cleavage order	No.	Specified	Semi-specified	Un-specified
**Leading**	24	0	3 (12.5)^b^	21 (87.5)^a^
**Lagging**	37	2 (5.4)^b^	2 (5.4)^b^	33 (89.1)^a^
**Total**	61	2 (3.3)^b^	5 (8.2)^b^	54 (88.5)^a^

^a,b^: values differ significantly at P<0.05

Lineage tracing of clones derived from leading blastomere (Fig 3, Table 2) revealed that none (0%) of the developed blastocysts could represent an orthogonal departure angle of fluorescent/non-fluorescent cells from the equatorial plane (specified pattern) and only 12.5% of developed blastocysts were classified as semi-specified. In contrast, majority of blastocysts (87.5%) represented great deviation in their angular degree (un-specified pattern).

Lineage tracing of clones derived from lagging blastomere revealed that 5.4% of developed blastocyst represented an orthogonal angular departure (specified pattern). Interestingly, for all the blastocysts in this category, the progeny of the lagging blastomere was located in embryonic part and we did not observe any blastocyst with absolute contribution of lagging blastomere to TE. Moreover, 5.4% of embryos were categorized as semi-specified based on their deviated angular departure. In contrast, majority of the blastocyst (91.9%) were classified un-specified based on the contribution of their lagging blastomeres to the Em-Ab regions (Fig 3, Table 2).

### Relative equality in developmental competence of corresponding sister blastomeres derived from ovine 2-cell embryos

**Fig 11:**
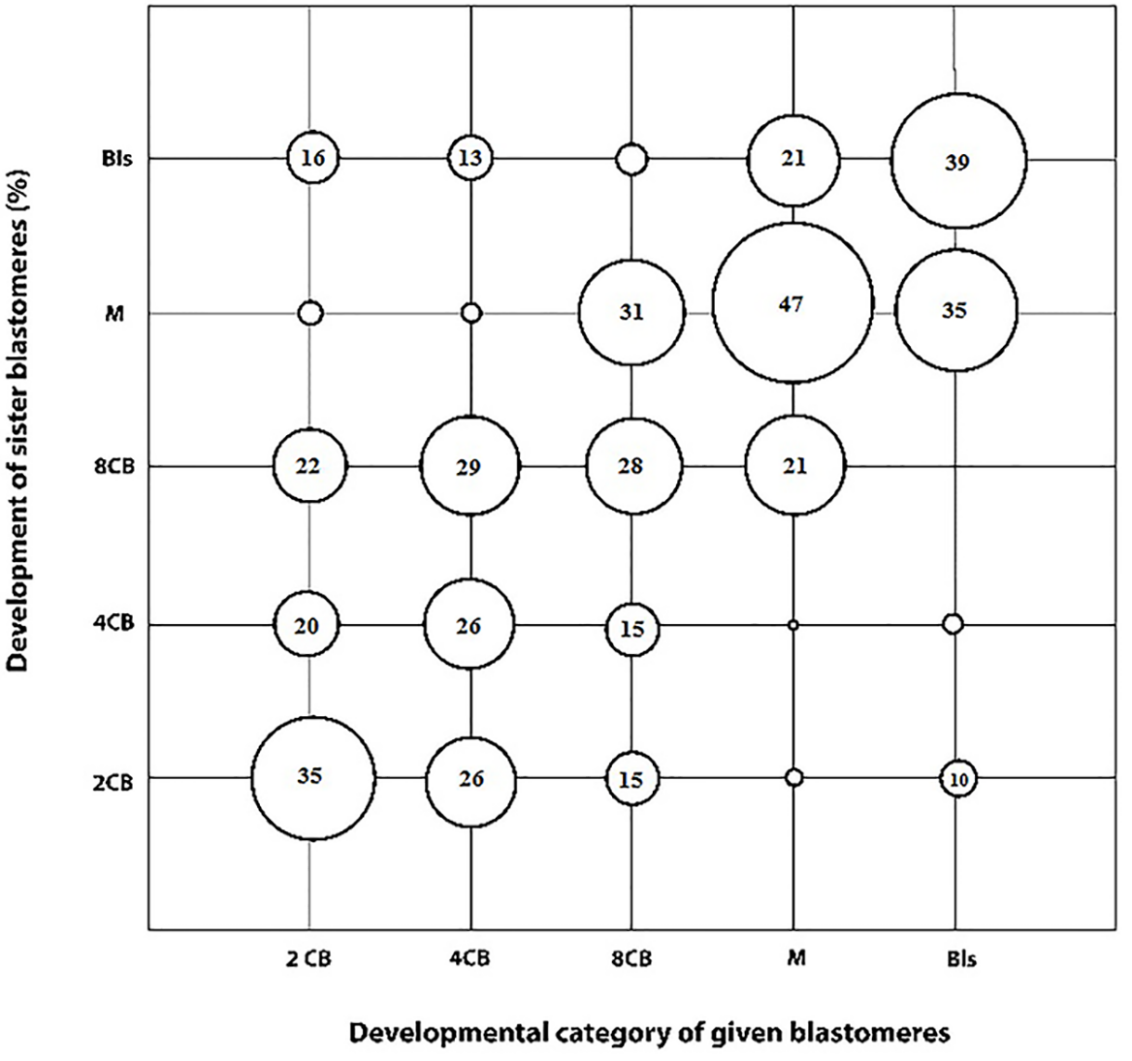
Developmental competence of corresponding sister blastomeres derived from ovine 2-cell embryos. The proportion of sister blastomeres that developed to certain developmental stages (y-axis: 2-cell block (2CB), two further cleavage (4-cell block: 4CB), three further cleavage (8-cell block: 8CB), four further cleavage (morula block: MB), and those blastomeres that reached the blastocyst stage (BLS)). The size of circles correspond their relative proportions (numbers within the circles).

A total of 456 IVF embryos at 2-cell stage from 6 replicates were used for sister blastomere separation. The average success rate for sister blastomere separation, evaluated by the ability to obtain two intact blastomeres, was over quite high (>93%). Pilot study showed that in vitro development of zona-free 2-cell stage embryos was non-significantly lower than zona-intact counterparts (31.5±8.6 vs. 36.7±5.9%, respectively). Fig 11 shows the relationship between developmental competence of a given blastomere to develop to a certain developmental stage (Y axis: 2CB, 2-cell block; 4CB, 4-cell block; 8CB, 8-cell block; MB: morula block; BLS, blastocyst) and its sister blastomere (X-axis: 2CB, 2-cell block; 4CB, 4-cell block; 8CB, 8-cell block; MB: morula block; BLS, blastocyst). As shown, the proportions of 2-cell stage ovine embryos in which both sister blastomeres stopped development before the blastocyst stage (2CB, 4CB, 4CB, and MB) or reached to the blastocyst stage (BLS) were 35.5, 26.3, 28.5, 46.8, and 39.6%, respectively. The probability that one sister blastomere stopped development before the blastocyst stage (at 2CB, 4CB, 4CB, or MB stage) while the other sister blastomere reached to the blastocyst stage was 16%. The cumulative probability that one blastomere developed one or more stage/s further than the corresponding sister blastomere was 43.9%. Likewise, the cumulative probability that one blastomere developed one or more stage/s before than the corresponding sister blastomere was 36.1%.

## Discussion

Oocyte polarity and embryonic patterning are well-established features of development in lower species [1]. Whether a similar form of pre-patterning exists in mammals is currently under hot debate in mice [50]. In porcine, two studies provided evidences of embryo patterning in relation with the biased distribution of cytoplasmic lipids and mitochondria between the 2-cell blastomeres [34, 35]. For the first time in ovine, we provided evidence of spatial distribution of total and specific transcripts and proteins within unfertilized MII oocytes. Importantly, the direction and orientation of the spatial arrangement of transcripts and proteins were both according to the MII-spindle position and clearly identified two hemispheres relatively comparable to that seen *Xenopus* oocytes. Analysis of fertilized oocytes also showed a clear bias in the preferential site of sperm entry into the ovine MII-oocytes. These results in agreement with some studies in mice [4, 51, 52] may suggest the existence of an animal-vegetal axis within the mammalian oocytes. Nonetheless, no significant difference was observed between the transcript abundances of 2-cell blastomeres, indicating that transcriptional polarity of unfertilized oocytes did not persist following fertilization. Therefore, it is necessary to distinguish between a possibly temporal polarization of maternal mRNAs and proteins within unfertilized oocytes of ovine oocytes and the genuine polarity that seen in oocytes of lower species such as *Xenopus* oocytes [33]. In contrast to the pre-patterning hypothesis, we did not observed any relationship between the order of division of 2-cell blastomeres and the pattern of cell allocation in ICM and TE of ovine blastocysts. Moreover, relative equality was observed between developmental competence of a given blastomere of 2-cell embryos and its sister blastomere to develop to a certain developmental stage in vitro. Therefore, while transcript and protein asymmetry within unfertilized oocyte provided evidences in support of pre-patterning in mammals, linages tracing of early cleaved blastomeres provided evidences against this hypothesis.

The membrane-soluble fluorescent dye Dil has been used for cell-lineage tracing of the 2-cell stage embryos [9]. For ovine 2-cell stage embryos, however, we could not label embryos by cell-membrane positioning of the dye. Unlike in mice, oocytes and embryos of ungulates have an opaque to dark appearance due to their characteristic high accumulation of lipid droplets and granules [34, 35, 40]. This lipid enriched cytoplasm may preclude simple absorption of oil dissolved Dil by the plasma membrane. Therefore, intracytoplasmic injection of Dil was used for labeling of two-cell ovine embryos which is consistent with the study of Park et al. [35] in porcine. Obtained results showed that although the overall developmental rate of injected embryos was lower than non-injected embryos, the likelihood of labeled and non-labeled blastomeres to divide first was equal, suggesting that the division order is independent of the labeling method.

In 75% of fertilized oocytes, sperm entry point was located in the meiotic spindle hemisphere. This rate is higher than a random rate of 50%. Importantly, the distribution of SEP in MII-half was not random as any (0%) sperm head was observed in zone-I which comprises 16.7% of the closest area to the meiotic spindle. This observation is consistent with the studies of Hiiragi and Solter [16] and Motosugi et al. [18] in mice. SEP was unrelated to the presence of cumulus cells because the same results were obtained when cumulus-corona cells were dispersed before IVF (data not shown). Therefore, the preferential SEP observed in ovine zygotes is reminiscent of the theory of the intrinsic polarity of mammalian oocytes which claimed by a number of researchers including Gardner et al. [8] and Piotrowska and Zernicka-Goetz [10]. These investigators demonstrated that the first cleavage plane and embryonal axis in mice can be defined with regard to the location of 2-pb and SEP. In this scheme, the blastomere in the 2-cell embryo that inherits SEP will divide earlier than the other and contribute preferentially to the ICM lineage [10]. In contrast, other researchers believe that mouse oocytes are asymmetric in the distribution pattern of cytoplasmic components and this provides no clues for the polarity of the oocyte or preferential SEP [16]. Noteworthy, Motosugi et al. [18] demonstrated that 2-pb moves toward the first cleavage plane and the preferential SEP to MII-half is due to space asymmetry of perivitelline space beneath the 1-pb. Accordingly, the probability of SEP was essentially random after zona removal, suggesting that there is no inherit preference for SEP in mouse oocytes [18]. Moreover, by rotation of the embryo within the zona, Kurotaki et al. [26] provided evidence showing that both the boundary between two-cell blastomeres and the Em-Ab axis of the blastocyst align relative to the ellipsoidal shape of zona pelucida. We also observed unbiased SEP in ovine oocytes when zona was removed before IVF (data not shown) which suggest the absence of preferential SEP in ovine oocytes. Further studies are essential to unravel the role of zona, perivitelline space and SEP in zygote of ovine and other species.

Compared to other mammalian species, oocytes and embryos of farm animals species are characterized by high lipid contents stored mainly as lipid droplets in the cytoplasm [53]. Apart from their role in energy metabolism during oocyte maturation and embryo development [54], asymmetric distribution of lipid droplets are postulated to be involved in lineage allocation in *Sminthopsis macroura* embryos [55]. A bias in in the lipid content of porcine 2-cell stage embryos was observed by Kim et al. (2012) [34]. The latter group demonstrated that blastomeres arising from lipid enrich blastomeres were 2-fold more likely to form the embryonic part than abembryonic part, while the contribution of brighter blastomeres (lipid-less) was just the opposite [34]. We observed that in majority of the 2-cell stage ovine embryos, the relative mean fluorescence intensities of both blastomeres were comparable and only in 11.1% of the embryos assessed a significant bias was observed in the distribution of lipid droplets between the two blastomeres. Lineage tracing of lipid-enriched and lipid-less blastomeres did not provide apparent correlation between lipid content and embryonal axis formation in ovine two-cell stage embryos (data not shown). Species-specific difference may be involved in this controversy.

While transcriptome regionalization is an essential polarity determinant among metazoans [4], one important question is whether these gene products stored symmetrically throughout the oocyte or the same transcript asymmetry of oocytes in lower specie exists within unfertilized mammalian eggs. To address this question, we compared the transcript abundances of genes involved in pluripotency and epigenetic reprogramming between 3 sub-cellular structures prepared microsurgically from ovine MII-oocytes: cortical materials containing MII-spindle material (S) and oocyte halves that either near-to (NS) or far-from (FS) the MII-spindle. A first point highlighted was that the overall mRNA content of FS halves was significantly higher than either NS or S but quite comparable to NS+S, suggesting symmetrical gradients of mRNA between two halves of MII-oocyte. Even though, since S compartments comprise a minute volume (≈2–5% of intact oocyte volume), the proportional amount of maternal mRNA restricted to the S is in range of 10-to-80 fold of FS, reflecting a clear bias in the spatial partitioning of maternal mRNA enrichment in association with MII-chromosomes in ovine. Interestingly enough, quantitative comparison of transcripts abundances revealed S, NS, and FS compartments of MII-oocyte have distinguishable mRNA profiles. Almost all the transcripts that were predominantly abundant in S or NS compartments belonged to genes involved in epigenetic regulation of pluripotency and imprinting. These results are compatible with a role for the transcriptional reorganization of mRNA sorting as a prelude to the establishment of cell fate later at the blastocyst [4, 7, 56].

Using microarray analysis of paired microsurgical spindle and remnant samples from mouse oocytes and zygotes, VerMilyea et al. [4] demonstrated that unfertilized MII-oocytes possess distinguishable profiles. Importantly, they demonstrated that while a significant bias in transcriptome profiles of zygote and associated spindle-enriched second polar body was still distinguishable after fertilization, no such transcriptional asymmetry was persist between sister cells within 2 or 3 cell embryos. In the same way, Robert et al. [57] demonstrated that although transcript content varied between both individual embryos and twin blastomeres, no consistent asymmetries were observed for the majority of genes. Therefore, in agreement with these two latter studies, we observed that the initial transcriptional asymmetry exist in the MII-oocyte may not persist through the early zygote and embryo cleavages. How the transcriptome asymmetry which we observed within ovine MII-oocytes (this study) and within mouse MII-oocytes and zygotes [4] could be eliminated within the first embryo cleavage? Although the exact answer remains to be understood, it may be related to the orientation of the first and second embryonic divisions, equatorial or meridional, along the animal-vegetal axis, with reference to MII-spindle as the hypothetical animal pole (Fig 3). A result of equatorial first cleavage division is the transcriptome asymmetry between the balstomeres of 2-cell embryo that could persist even after the second cleavage division. By contrast, a result of meridional first cleavage division is the transcriptome symmetry between the balstomeres of 2-cell and 3-cells embryos, despite oocyte transcript polarity.

In lower vertebrates and invertebrates, the polarity of an embryo which underlies the future body plan is anticipated before fertilization by polar distribution of protein domains and their concentration gradients within the egg [58]. The spatial distribution of proteins within the animal and vegetal poles of amphibian oocytes/eggs has been also documented [59, 60]. However, mammals appear to be exception to this. For example in mouse, oocyte is thought to be “asymmetric” but “non-polarized” [18]. In the same sense, the embryo polarity is generally thought to be established after implantation not before/during fertilization [16,18]. Even though, some studies believe that the same model of oocyte polarity of amphibians exists in mammals, and therefore, the embryo polarity could be traced back to the organization of the egg [51, 61, 52, 62]. So far, there are no studies which have investigated possible total protein regionalization in mammals, except for few studies that have described the spatial rearrangement of some unique proteins within mouse oocyte [25, 62]. We described here that maternal proteins are spatially partitioned within unfertilized ovine oocytes and they are highly restricted to the MII-chromosomes. Importantly, immunoblotting revealed that specific maternal proteins such as DNMT3A and NANOG were also asymmetrically enriched in MII-spindle-half of the oocytes. These results may provide the first preliminary evidence for the notion that pivotal events of early mammalian development, such as embryonal axis formation and first lineage commitment, may be influenced by the stockpile of maternal proteins which have been inherited by the oocyte prior to fertilization.

Lineage-tracing of ovine labeled embryos demonstrated that while the progeny of fast 2-cell blastomere contributed to more cells in the developed blastocysts compared to lagging blastomeres, the progenies derived from the leading/fast and lagging/slow blastomeres do not have a specific fate and their allocation do not delineate the embryonic-abembryonic polarity of the blastocyst. These data are in contrast with the related studies in porcine [34, 35] and mouse [5, 8, 9, 10, 11] but are in agreement with some other infield studies in the mouse [12, 13, 15, 16, 17 18]. Importantly, relative equality was observed between developmental competence of a given blastomere of 2-cell embryos and its sister blastomere to develop to certain developmental stages in vitro (2-cell block, 4-cell block, 8-cell block, morula block, or blastocyst). In a similar study in bovine, Held et al. [46] showed an overall correlation coefficient of 73% in terms of in vitro development of bisected bovine sister blastomeres. Even though, species-specific differences in the mechanism of embryo development may prevent a direct comparison between these studies, and therefore, an implicit assumption is that each species should be considered separately from the other species.

The most important factor affecting the oocyte competence is the legacy from the follicular environment prior to meiotic resumption which provides a full support during early embryonic events until broad embryonic genome activation [63]. In the current model of mammalian oogenesis, it is though that this huge maternal store is generated intrinsically when the oocyte is reaching its full size within the follicle [64]. However, very recently Macaulay et al. [65] provided evidence of a synapse-like vesicular trafficking connection which support selective transfer of large molecules, such as transcripts, from cumulus cells into the fully grown bovine oocyte. Considering this unexpected contribution of cumulus cells to the maternal stores, it would be interesting to know whether the observed polarities of transcripts [4, 57] and proteins (this study) within mammalian oocyte could be linked to the same levels of functional polarity of the surrounding cumulus cells.

Classically, following blastomere separation of 2-cell stage embryo, each blastomere can often develop to the blastocyst and to the term [66, 6, 15, 29, 67, 68, 69]. This high degree of developmental plasticity lends support to suggest that that early embryonic development in mammals, in contrast to development of other species, is stochastic [30]. Knowledge of the developmental plasticity with which embryos adapt to experimental perturbation offers promising opportunities to propagate animals with similar genetic background for research, biomedicine and agriculture. Yet, there are indications in earlier and recent works that denying the universality of developmental plasticity provided that monozygotic twining by this method of embryo splitting is practically unattainable [29, 30, 70, 71]. That could be explained by the findings that even if separated blastomeres of 2-cell embryo could develop to the blastocyst, very few or neither pregnancy may be resulted in term birth of monozygotic twins by the route, suggesting the biased developmental competence of 2-cell blastomeres [29, 30]. Even though, owing unbiased contribution of ovine 2-cell blastomeres established in this study and earlier work on monozygotic twining in ovine [67], one may argue that early embryo development in ovine, in contrast to lower animals such as *Xenopus* and *Drosophila* is both stochastic and regulative.

### Conclusion

To the best of our knowledge, this study is the first to describe a polarized distribution of some maternal transcripts and total maternal proteins in ovine unfertilized oocytes. These findings may propose that some pivotal events of early mammalian development, such as embryonal axis formation and first lineage commitment, may be influenced by the stockpile of maternal proteins which have been inherited by the oocyte prior to fertilization. These results may be relevant to low developmental competence of SCNT embryos in which such a great source of maternal mRNAs and proteins are removed during oocyte enucleation. Moreover, refinements may need to be considered in some manipulative and diagnostic techniques. Perhaps for example, developmental viability of embryos after ICSI may be clinically relevant with the place of sperm injection; provided that ovine matured oocytes have a preferential sperm entry very close to maternal chromosomes, though that this tendency was found to be associated to the zona. Even though, we observed evidences of transcript symmetry and unbiased contribution of 2-cell blastomeres to the ICM and TE. Moreover, relative equality was observed between developmental competence of a given blastomere of 2-cell embryos and its sister blastomere to develop to certain developmental stages. These data along with other data of comparable developmental competence of 2-cell blastomeres may highlight the potential usage and clinical relevant of the sister blastomere of bisected 2-cell embryo for monozygotic twining [30, 31], autologous ESC-establishment [30], and prediction of the developmental competence of the corresponding sister blastomere [46]. Therefore, even if unfertilized ovine oocyte could be considered polar with respect to spatial regionalization of maternal mRNA and proteins, the principle forces of this definitive polarity axis may not persist during embryonic cleavages. In this sense, embryo development and embryonal axis formation could not be traced back to events before fertilization.

## Supporting Information

S1 Fig RNA quality assessment. To assess the integrity of total RNA, an aliquot of each RNA sample was run on a denaturing agarose gel stained with ethidium bromide. Intact total RNA run on a denaturing gel had sharp, clear 28S and 18S rRNA bands, indicating the RNA quality.
(TIF)

S2 Fig The specificity of antibodies. Immunoblotting was performed on ovine tissues (testis and liver) and fibroblasts. Obtained data indicated that among seven antibodies (SOX2, DNMT3A, DNMT3B, DNMT1, cKIT, OCT4, and NANOG) that were checked for cross-reactivity with the corresponding ovine proteins. Only DNMT3A, DNMT3B and NANOG antibodies reacted with the corresponding ovine proteins.
(TIF)

S1 Table The relationship between time interval post insemination (hpi) and fertilization and sperm chromatin status in ovine eggs. (DOCX)

S2 Table Topological relationship between the MII-spindle and the 1-PB in ovine oocytes (n = 268). (DOCX)

S1 Video Microsurgical trisection of ovine unfertilized MII-oocytes using a manual method of oocyte trisection. (AVI)
