# Supplementary figures and images for: The Principal Forces of Oocyte Polarity Are Evolutionary Conserved but May Not Affect the Contribution of the First Two Blastomeres to the Blastocyst Development in Mammals
Source: PLoS One. 2016 Mar 31;11(3):e0148382. doi: 10.1371/journal.pone.0148382 (PMC4816511; doi:10.1371/journal.pone.0148382)

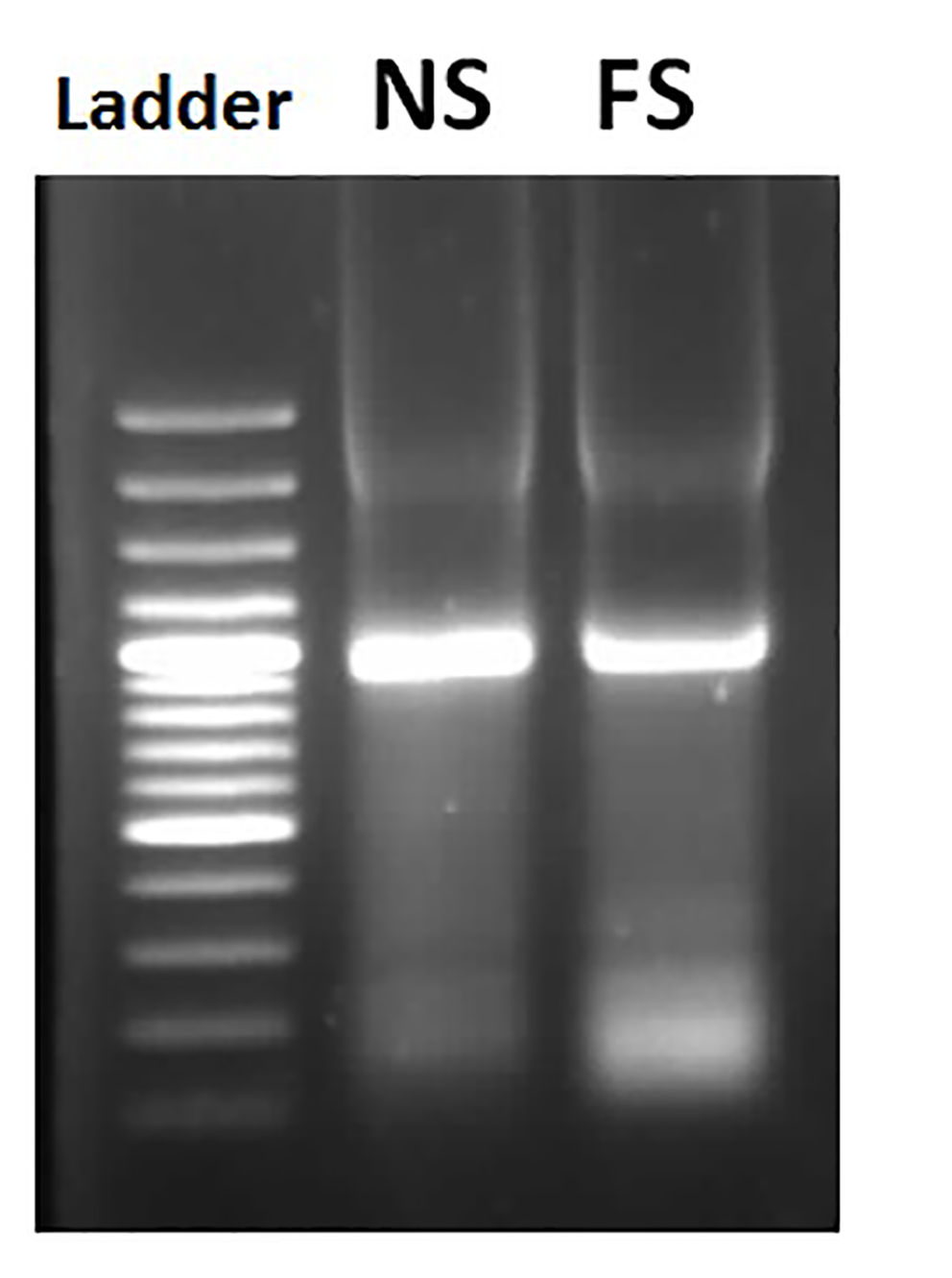

Supplement: S1 Fig — To assess the integrity of total RNA, an aliquot of each RNA sample was run on a denaturing agarose gel stained with ethidium bromide. Intact total RNA run on a denaturing gel had sharp, clear 28S and 18S rRNA bands, indicating the RNA quality. (TIF) [file pone.0148382.s001.tif]

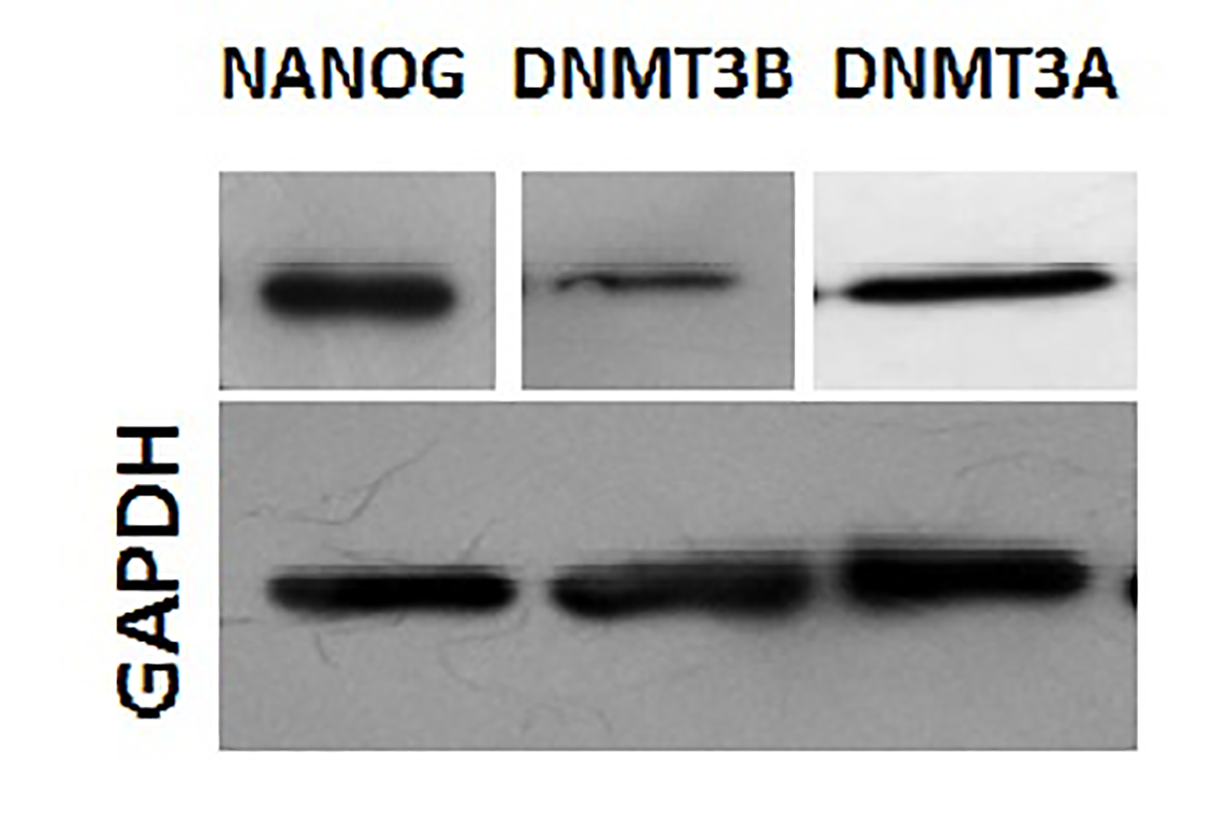

Supplement: S2 Fig — Immunoblotting was performed on ovine tissues (testis and liver) and fibroblasts. Obtained data indicated that among seven antibodies (SOX2, DNMT3A, DNMT3B, DNMT1, cKIT, OCT4, and NANOG) that were checked for cross-reactivity with the corresponding ovine proteins. Only DNMT3A, DNMT3B and NANOG antibodies reacted with the corresponding ovine proteins. (TIF) [file pone.0148382.s002.tif]
