# Supplementary material for: The Principal Forces of Oocyte Polarity Are Evolutionary Conserved but May Not Affect the Contribution of the First Two Blastomeres to the Blastocyst Development in Mammals
Source: PLoS One. 2016 Mar 31;11(3):e0148382. doi: 10.1371/journal.pone.0148382 (PMC4816511; doi:10.1371/journal.pone.0148382)
Supplement: S1 Table — (DOCX) [file pone.0148382.s003.docx]

| Supp. Table 1. The relationship between time interval post insemination (hpi) and fertilization and sperm chromatin status in ovine eggs. | | | | | | |
| --- | --- | --- | --- | --- | --- | --- |
|  |  |  |  | Sperm chromatin status | | |
| hpi | n | Fertilized (%) |  | Intact (%) | PCC (%) | PN (%) |
| 3 | 43 | 22 (50.6) |  | 22 (100.0)a | 0 (0.0) b | 0 (0.0) c |
| 4 | 37 | 27 (74.3) |  | 27 (100.0)a | 0 (0.0) b | 0 (0.0) c |
| 5 | 40 | 34 (86.3) |  | 30 (88.1)a | 4 (11.9)ab | 0 (0.0) c |
| 6 | 31 | 26 (83.5) |  | 23 (89.4)a | 1 (4.3)b | 2 (8.7) b |
| 7 | 46 | 41 (89.3) |  | 37 (90.3)a | 1 (2.4)b | 3 (7.3) b |
| 8 | 35 | 30 (87.1) |  | 25 (83.7)a | 2 (6.7)b | 3 (10.0) b |
| 12 | 30 | 27 (90.3) |  | 10 (37.0)b | 5 (18.5)a | 12 (44.4) a |
| a-c: within each column, values with different letters differ significantly at P<0.05. | | | | | | |
