# Supplementary material for: The Principal Forces of Oocyte Polarity Are Evolutionary Conserved but May Not Affect the Contribution of the First Two Blastomeres to the Blastocyst Development in Mammals
Source: PLoS One. 2016 Mar 31;11(3):e0148382. doi: 10.1371/journal.pone.0148382 (PMC4816511; doi:10.1371/journal.pone.0148382)
Supplement: S2 Table — (DOCX) [file pone.0148382.s004.docx]

| Supp. Table 2. Topological relationship between the MII-spindle and the 1-PB in ovine oocytes (n=268). | | |
| --- | --- | --- |
| Zone* | MII-spindle angle  with 1-PB | n (%) |
| Zone-I | 0-5° | 42 (15.7^b^) |
| Zone-II | 6-45° | 115 (42.9^a^) |
| Zone-III | 46-90° | 105 (39.2^a^) |
| Zone-IV | >90° | 6 (2.2^c^) |
| *Zones I-III represent the MII-oocyte half and zona-IV represents the non-MII oocyte half.  ^a-c^: Values with different letters are significantly different at P≤0.05. | | |
